# Direct Z-scheme heterojunction impregnated MoS_2_–NiO–CuO nanohybrid for efficient photocatalyst and dye-sensitized solar cell

**DOI:** 10.1038/s41598-024-65163-5

**Published:** 2024-06-24

**Authors:** Karthigaimuthu Dharmalingam, Arjun Kumar Bojarajan, Ramalingam Gopal, Elangovan Thangavel, Salah Addin Burhan Al Omari, Sambasivam Sangaraju

**Affiliations:** 1 Smart Energy Materials Research Laboratory, Department of Energy Science and Technology, Periyar University, Salem, India; 2 Department of Mechanical and Aerospace Engineering, United Arab Emirates University, Al Ain, 15551 UAE; 3 National Water and Energy Center, United Arab Emirates University, Al Ain, 15551 UAE; 4 Quantum Materials Research Lab (QMRL), Department of Nanoscience and Technology, Alagappa University, Karaikudi, India

**Keywords:** Materials for energy and catalysis, Nanoscale materials

## Abstract

In this present work, the preparation of ternary MoS_2_–NiO–CuO nanohybrid by a facile hydrothermal process for photocatalytic and photovoltaic performance is presented. The prepared nanomaterials were confirmed by physio-chemical characterization. The nanosphere morphology was confirmed by electron microscopy techniques for the MoS_2_–NiO–CuO nanohybrid. The MoS_2_–NiO–CuO nanohybrid demonstrated enhanced crystal violet (CV) dye photodegradation which increased from 50 to 95% at 80 min; The degradation of methyl orange (MO) dye increased from 56 to 93% at 100 min under UV–visible light irradiation. The trapping experiment was carried out using different solvents for active species and the Z-Scheme photocatalytic mechanism was discussed in detail. Additionally, a batch series of stability experiments were carried out to determine the photostability of materials, and the results suggest that the MoS_2_–NiO–CuO nanohybrid is more stable even after four continuous cycles of photocatalytic activity. The MoS_2_–NiO–CuO nanohybrid delivers photoconversion efficiency (4.92%) explored efficacy is 3.8 times higher than the bare MoS_2_ (1.27%). The overall results indicated that the MoS_2_–NiO–CuO nanohybrid nanostructure could be a potential candidate to be used to improve photocatalytic performance and DSSC solar cell applications as well.

## Introduction

Energy and environmental concerns are two primary concerns in our daily lives. The scarcity of fossil fuels and the release of untreated toxic waste into the ecosystem has contributed to a dramatic increase in energy and environmental problems in recent years^1^. Dye-sensitized solar cells (DSSC) solar cells and photocatalytic dye degradation employing semiconductor materials are recognized as among the most promising technologies to generate sustainable energy from abounding resources. Although several DSSCs and photocatalysts have been established over the last few decades, they are still far from satisfying the efficiency and stability demands of industrial customers. The most significant obstacle is to create a photocatalyst that is highly effective, inexpensive, and eco-friendly, as well as a DSSC that is capable of achieving the requirements of practical applications^2^. Developing a heterojunction between various semiconductor phases provides an efficient approach for increasing the separation of photogenerated electrons and hole pairs. In terms of heterojunctions, the most prevalent varieties are the P-N, non-P-N, and Z-scheme heterojunctions. Among these, the Z-scheme heterojunction was initially proposed by Bard in 1979^3^. The design and construction of Z-scheme heterojunction exist in a high number of shapes and always depend on the synthesis procedures. However, Z-scheme heterojunction characterizations provide information about the morphology of a remarkable number of phases, crystallographic defects, different phases, and chemical composition^4^. Therefore, various morphological characterization techniques offered, among all nanospheres with Z-scheme heterojunction are excellent and have been extensively explored because they may be modified over a broad variety of material compositions. In this aspect, morphologies of Z-scheme heterojunction influence the various properties such as, optical, electrical, electronic, magnetic, and thermal. It can be used in technological development for various applications^5,6^.

Molybdenum Disulfide (MoS_2_) has been used as a promising and prospective material due to its novel structure as well as unique electrical and optical characteristics. Especially, it has also been used as a platinum free counter electrode (CE) in DSSC and as a catalyst for photocatalytic activity^7–9^. Recent research has focused on the combination of MoS_2_ with inorganic compounds such as metal sulfides, metal oxides, nitrides, and carbides to increase photocatalytic activity and DSSC solar cell performance via the use of simple production procedures. The morphological characterization based transition-metal oxides, such as RuO_2_, In_2_O_3_, Ag_2_O, CeO_2_, WO_3_, and SnO_2_ have caused a lot of attention, but these materials are costly and very harmful which prevents them from being used in photovoltaic and photovoltaic applications. Among all, the transition metal oxides, Nickel Oxide (NiO) and Copper Oxide (CuO) are potential candidates in the field of photovoltaic and photocatalytic applications due to their favorable electrical structure, excellent light absorption expression, stable redox ability, and low band gap^10,11^. An advantage of the catalytic reactions of these materials is the rapid formation of redox pairs on the surface of the active material, which improves their efficiency by oxidizing them^12^. For example, the electrocatalytic HER activity was carried out by Lin and colleagues using a composite containing MoS_2_/TiO_2_/NiO and it has a low overpotential with strong HER activity. Due to the closed interface bonding between the monolayer MoS_2_ and TiO_2_, with the addition of NiO^13^. The MoS_2_/CdSe/NiO photoelectrode, constructed by Yuming Dong et al., outperformed the standard CdSe/NiO electrode in photocatalytic and photoelectrochemical studies. The standard deviation of the hydrogen evolution rates on a fabricated photocathode in a pH 6 buffer solution was 0.52 µmol h^−1^ at − 0.131 V, with about 100% faradaic efficiency^14^. Mehboob et al. developed a NiO and MoS_2_ heterojunction for photoelectrochemical (PEC) water splitting and removal of bromophenol blue (BPB) by visible light. The strong 2D/2D interactions have great interfacial coupling, which increases activity. With a maximal photocurrent density of 1.57 mA/cm^2^ at a lower potential, NiO (15.0%) and photocatalytic exhibited 87% of photodegradation. A simple hydrothermal method was used for developing the MoS_2_@CuO composite by Honglin Li et al., which revealed the hetero nanoflower morphology. Methylene blue (MB) dye was used to dramatically increase the MoS_2_@CuO heterojunctions photocatalytic activity, and after 100 min, there was only around 4.3% of MB left in the solution under UV–vis light^15^. Yunqi Wang et al. report the effective development of a Z-scheme MoS_2_/CuO photocatalyst via calcination-assisted hydrothermal method, which exhibits high activity (96%) in the removal of 2-mercaptobenzothiazole (MBT) when exposed to visible light^16^. Penghui Ye et al. fabricated a CuO@MoS_2_ core–shell by combining hydrothermal deposition with in situ engraving. The degradation of tetracycline (TC) via visible light was used to assess the photocatalytic activity of the produced photocatalyst. In only 120 min of exposure to visible light, 79% of the tetracycline was degraded via Z-scheme charge transfer of Cu@CuO@MoS_2_ core-shell^17^.

In the photovoltaic aspects, MoS_2_, NiO, and CuO have been shown to play a significant role, and notable DSSC research has been conducted through these materials. For example, Wun-Hao Jhang et al. fabricated the commercial MoS_2_ powder counter electrode for DSSC, which exhibited 1% of PCE^18^. Hui Wang et al. fabricated the DSSC with the CE consisting of commercial NiO powder, which exhibited a PCE of 0.28%^19^. Wongratanaphisan et al., reported that commercial copper powders based on CuO were prepared by the additive microwave heating technique for CE of DSSC, which exhibited 1.76% of PCE^20^. MoS_2_, NiO, and CuO have been used as counter-electrode materials for DSSC for many years, but no high-efficiency DSSC has been obtained yet from this pure, binary, and ternary hybrid. In addition, the efficient functioning requirement of a DSSC is highly dependent on the minimizing of charge recombination and the effective interfacial charge transport at the dye, electrodes, and electrolyte interfaces. This could be attained by constructing a counter electrode with an effective transport route for the photoinduced charge carriers to make their way to the current collector.

Besides, one of the most promising methodologies for achieving high efficiency of DSSC and photocatalyst is the engineering of Z-scheme heterojunctions formed by multiple semiconductor materials, respectively. In particular, by carefully selecting synergistically supplementary semiconductor materials for Z-scheme heterojunction, they may exhibit stronger physicochemical properties and overcome some drawbacks present in homojunction analogs, such as unsuitable band gaps and low light absorption ability^21,22^. Most of the charge carrier transfer intermediates in the Z-scheme heterojunction were found to be noble metals (such as MoS_2_, NiO, and CuO) in the previous studies. In this regard, when the semiconductor materials MoS_2_, NiO, and CuO are incorporated into the heterogeneous phase of the ternary hybrid, due to their unique features and tunable properties, the reaction provides high electron–hole pair separation and rapid charge transfer at the MoS_2_, NiO and CuO interfaces^11,23,24^, which will clear that the use of MoS_2_–NiO–CuO ternary composite can further improve the surface morphology and high efficiency of PCE% in DSSC.

In this present work, we synthesized a Z scheme heterojunctions based MoS_2_/NiO/CuO ternary hybrid, to serve as the photocatalyst and DSSC solar cell. The synthesized ternary hybrid showes enhanced photocatalytic under light irradiation and also fabricated the counter electrode of the DSSC solar cell. Finally, CuO with MoS_2_–NiO is said to be responsible for the improved DSSC solar cell and photocatalytic activities and their performances were discussed in detail.

## Experimental methods

### Reagents

Sodium molybdate dihydrate (Na_2_MoO_4_·2H_2_O, assay 99.5%), l-Cysteine (C_3_H_7_NO_2_S, assay 97%), Nickel(II) nitrate hexahydrate (Ni(NO_3_)_2_·6H_2_O, assay 98%), Ammonia solution (NH_4_OH), Copper (II) Nitrate Hexahydrate (Cu(NO_3_)_3_·6H_2_O, assay 98.5%), Methyl Orange (MO, C_14_H_14_N_3_NaO_3_S, assay 85%), Crystal Violet (CV, C_25_H_30_ClN_3_, assay 85%), 2-propanol (IPA, C3H8O, assay ≥ 99.8%), silver nitrate (AgNO_3_, assay ≥ 99.0%), and benzoquinone (BQ, C6H4O2, assay ≥ 98.0%), Poly Vinylidene Fluoride (PVDF, –(C_2_H_2_F_2_)n–, assay ≥ 99.6%), N-Methyl-2-Pyrrolidone (NMP, C_5_H_9_NO, 98% pure), Polyethylene oxide-Mw: 250,000 ((–CH_2_CH_2_O–)n, pure 99%), Polyethylene glycol (C_2n_H_4n+2_O_n+1_, > 99% pure), Di-tetrabutylammonium cis-bis(isothiocyanato)bis(2,2′-bipyridyl-4,4′-dicarboxylato)ruthenium(II) (N719 Ruthenium dye, C_58_H_86_N_8_O_8_RuS_2,_ assay > 97%), Lithium iodide (Lil, assay ≥ 99.0%), 1-propyl-2,3-dimethylimidazoliumiodide (C_8_H_15_IN_2_, 99% pure), and Acetonitrile (CH_3_CN, 99% pure) were purchased from Sigma Aldrich, India. All chemicals were used exactly as supplied and were of an analytical grade unless otherwise specified. The made in the laboratory deionized (DI) water was utilized in all experiments.

### Synthesis of MoS_2_

0.3 g of Na_2_MoO_4_·2H_2_O and 0.6 g of C_3_H_7_NO_2_S were dissolved in 30 mL of DI water separately and stirred. These solutions were mixed after 1 h of stirring, and the solution was sonicated for 5 min. The mixed solutions were transferred into a 100-mL autoclave and maintained at 200 °C for one day. The autoclave was naturally cooled and the dark blue precipitated sample was filtered, centrifuged, and washed with DI water and twice ethanol and dried at 80 °C for half a day. Finally, the MoS_2_ was obtained. The preparation of NiO and CuO was discussed in S.I.

### Synthesis of MoS_2_–NiO

To begin with, 0.6 g of Na_2_MoO_4_·2H_2_O and 1.2 g of C_3_H_7_NO_2_S were dissolved in 40 mL of DI water, and 0.4 g of the NiO as prepared was dissolved in 40 mL of DI water separately and stirred for 1 h. The stirred solution was then mixed drop by drop, and the above mixture was sonicated for 15 min. The mixed solution was transferred into a 100 mL autoclave and maintained at 180 °C for one day. The sample was filtered washed and dried at 80 °C for half a day. Finally, the MoS_2_–NiO composite is obtained.

### Synthesis of MoS_2_–NiO–CuO

In 40 mL of DI, 0.4 g of Na_2_MoO_4_·2H_2_O and 0.8 g of C_3_H_7_NO_2_S were dissolved, and subsequently, 0.25 g of the as-prepared NiO and 0.18 g of CuO were dissolved separately in 40 mL of DI water and stirred for 1 h. The stirred solution was then mixed drop by drop, and the above mixture was sonicated for 15 min. The mixed solution was transferred into a 100 mL autoclave and treated at 180 °C for one day. The sample was centrifuged, filtered, washed, and dried at 80 °C for half a day. Finally, the MoS_2_–NiO–CuO nanohybrid is obtained.

### DSSC fabrication

The doctor-bladed method has been used to prepare the two active electrodes. The prepared samples were mixed with PVDF (0.95:0.05) and a few drops of NMP solution to form a colloid used as the counter electrodes. After that, the colloid was deposited on the FTO glass. To prepare photoanode, the commercial TiO_2_ nanoparticles were mixed with Triton X-100, polyethylene oxide, and polyethylene glycol to create a TiO_2_ slurry paste. The TiO_2_ slurry was coated on the FTO plate and annealed at 500 ºC for 3 h and the photoanode was dipped with 3 mM N719 dye solution for one day. The gel electrolyte was prepared by using 0.6 M of 1-propyl-2,3-dimethylimidazolium iodide, 0.5 M of 4-tert-butylpyridine, 0.1 M of LiI, 0.05 M of I_2_, and 3% w/w of polyethylene oxide and 5 mL of acetonitrile with continuous stirring for half day^25^. Finally, the photoelectrode was accumulated with the as-prepared counter electrode convoyed with the injection to a few drops of electrolyte between the two electrodes and fabricated the complete DSSC device^7^.

The following formulas were used to determine the fill factor (FF%) and power conversion efficiency (PCE%),

1 $$FF = \left( {J_{max} \times V_{max} } \right)/ \left( {J_{sc} \times V_{oc} } \right)$$

2 $$PCE \;\left( \% \right) = \frac{{\left( {FF \times J_{sc} \times V_{oc} } \right)}}{{P_{in} }}$$

### Photocatalytic degradation

The photocatalytic experiment was conducted in a cylindrically shaped photocatalytic system under 500-W halogen light. To prepare the reaction solution, 0.02 g of sample and 20 ppm of methyl orange (MO) or crystal violet (CV) dye were mixed with 100 mL of DI water and this reaction solution was placed into the dark. The adsorption and desorption equilibrium was reached when the suspension was continuously stirred in a dark place for 1 h. After adsorption and desorption equilibrium processes, the dye solution was placed under 500-W halogen light. While UV–visible light irradiation on the dye solution, 3 mL of suspension is continuously withdrawn at 30 min intervals and the solution is centrifuged. The concentration of the recovered centrifuged dye solution was then measured using a UV–vis spectrometer. The degrading efficiency, first order kinetics and second order kinetics of the photocatalyst was calculated using the following equations.

3 $${\text{Degradation }}\;{\text{efficiency}}\;(\% ) = \left( {\frac{{{\text{C}} - {\text{C}}_{0} }}{{\text{C}}}} \right) \times 100\%$$

4 $$\ln \frac{{C_{0} }}{C} = - k_{1} {\text{t}}$$

5 $$\frac{1}{C} = \frac{1}{{C_{0} }} + k_{2} t$$

Here, C and C_0_ signified initial and final concentrations of the dye, k_1_ is the pseudo-first-order rate constant (min^−1^) and k_2_ is the pseudo-second-order rate constant (M^−1^ min^−1^), respectively. The stability test was carried out four times, each time maintaining the same settings as the previous photocatalytic experiment.

### Scavengers test

The active species most strongly associated with the degradation process are also identified using a trap test. For the same experiment described above, the three kinds of 1 mmol scavengers for hydroxyl (OH), electrons (e^−^), and superoxides (^**·**^O_2_^−^) radicals were taken: 2-propanol (IPA), silver nitrate (AgNO_3_), and benzoquinone (BQ). After the first run was finished, the photocatalyst was removed from the dye suspension and repeatedly rinsed with DI water. The photocatalyst was then employed in the next cycle after being dried at 80 °C for 8 h. To determine the stability of the catalyst, four cycles of photocatalytic tests were conducted and the remaining catalyst was studied by XRD and SEM analysis.

### Characterizations

The crystal phase purity of the obtained samples was examined by a Rigaku Miniflex X-ray diffractometer equipped with a Cu-Kα radiation source (λ = 1.5406 Å). The FTIR spectrum of obtained samples was measured by Brucker Tensor 27 spectra. The Phi Versaprobe III type of X-ray photoelectron spectrometer was used to examine the elemental composition of the prepared samples. Ultraviolet and visible absorption diffuse reflectance spectra (UV–Vis-DRS) and photoluminescence (PL) studies were recorded by the Perkin Elmer Lambda 25 and FP-8200 fluorescence spectrometers. A Carl Zeiss (USA) model scanning electron microscope (SEM) attached to an EDAX detector and a JEOL JEM-2100 model transmission electron microscope (TEM) working at 200 kV were used to determine the morphology of the obtained samples. The thickness of the DSSC was measured using the SJ-301 Mitutoyo Surface Profilometer. The Scientech (SS 50 K, AAA)-EM employing an A.M. 1.5 G solar simulator with a Keithley 2400 m was utilized to investigate the characterizations of the fabricated DSSC. A PG-LYTE 1.0 electrochemical workstation was used for determining the photocurrent correspondence of the fabricated DSSC.

## Results and discussion

### XRD analysis

**Figure 1:**
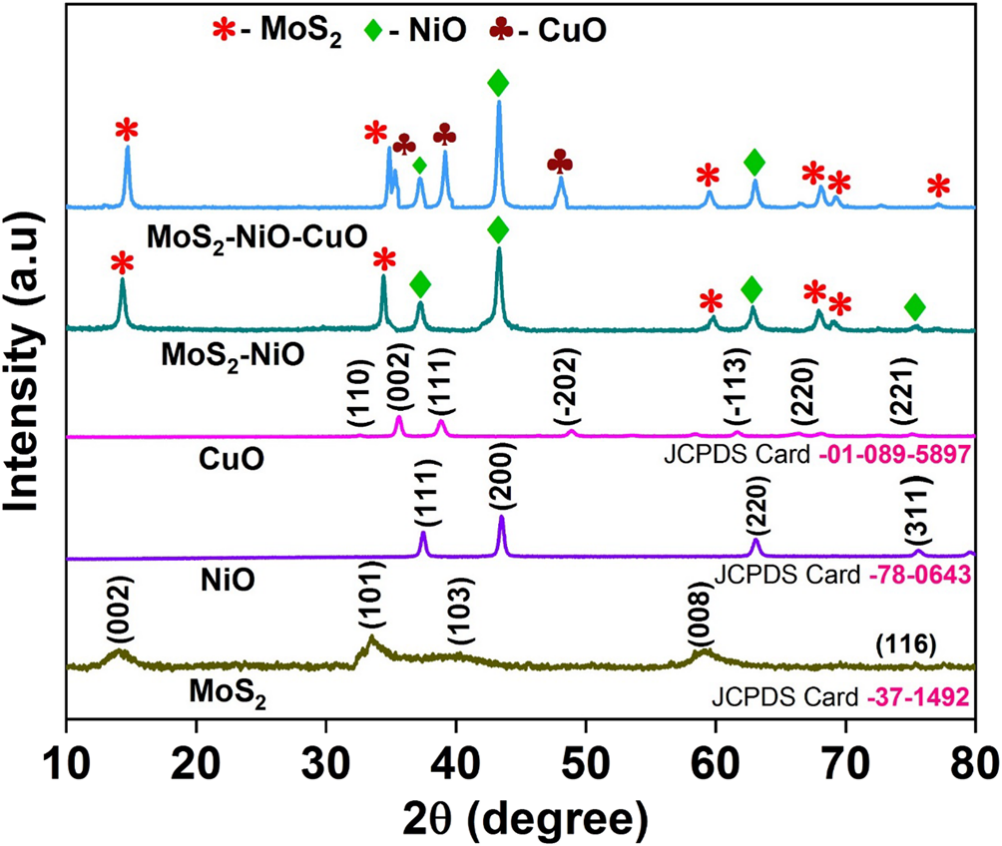
XRD patterns of pure MoS_2_, NiO, CuO, MoS_2_–NiO and MoS_2_–NiO–CuO nanohybrid.

The XRD patterns were used to investigate the crystal phase growth and purity of the obtained samples, as shown in Fig. 1. The crystal planes (002), (100), (110), (008) and (116) of MoS_2_ were represented by the diffraction (2θ) peaks at 14.3°, 33.3°, 40.20°, 59.21° and 75.90° respectively. The XRD patterns of MoS_2_ were accurately assigned to its hexagonal phase structure by comparing them to JCPDS PDF No. 37-1492^26^. The (111), (200), (220), and (311) reflection planes of pure NiO and MoS_2_–NiO, respectively, were ascribed to the diffraction peaks at 2θ = 37.42°, 43°, 63.04°, and 75.40° (JCPDS Card No. 78-0643)^27^. The CuO peaks at 32.54°, 35.71°, 38.86°, 48.89°, 61.66°, 68.09° and 72.55° ascribed to the (110), (002), (111), ($\overline{ 2}$02), ($\overline{ 1}$13), (220) and (221) planes with JCPDS Card 01-089-5897^28^. The presence of the contributing peaks for MoS_2_, NiO, and CuO in the MoS_2_–NiO–CuO nanohybrid diffraction peaks demonstrates the successful fabrication of the ternary MoS_2_–NiO–CuO nanohybrid. However, the diffraction peak positions of pure MoS_2_ and MoS_2_–NiO–CuO nanohybrid were compared, showing that the 2θ values of the prepared MoS_2_–NiO–CuO nanohybrid changed from 0.2° to 1.2°. In particular, the diffraction peak at 75.9° in MoS_2_–NiO–CuO is shifted to 77.04° due to the incorporation of CuO is higher in MoS_2_ than NiO. After the formation of MoS_2_–NiO–CuO ternary nanohybrid, the peak intensity of MoS_2_ in MoS_2_–NiO–CuO nanohybrid was enhanced, indicating the influences of CuO in MoS_2_–NiO. The discussion of the elemental mapping stated before is supported by this information.

### FTIR analysis

**Figure 2:**
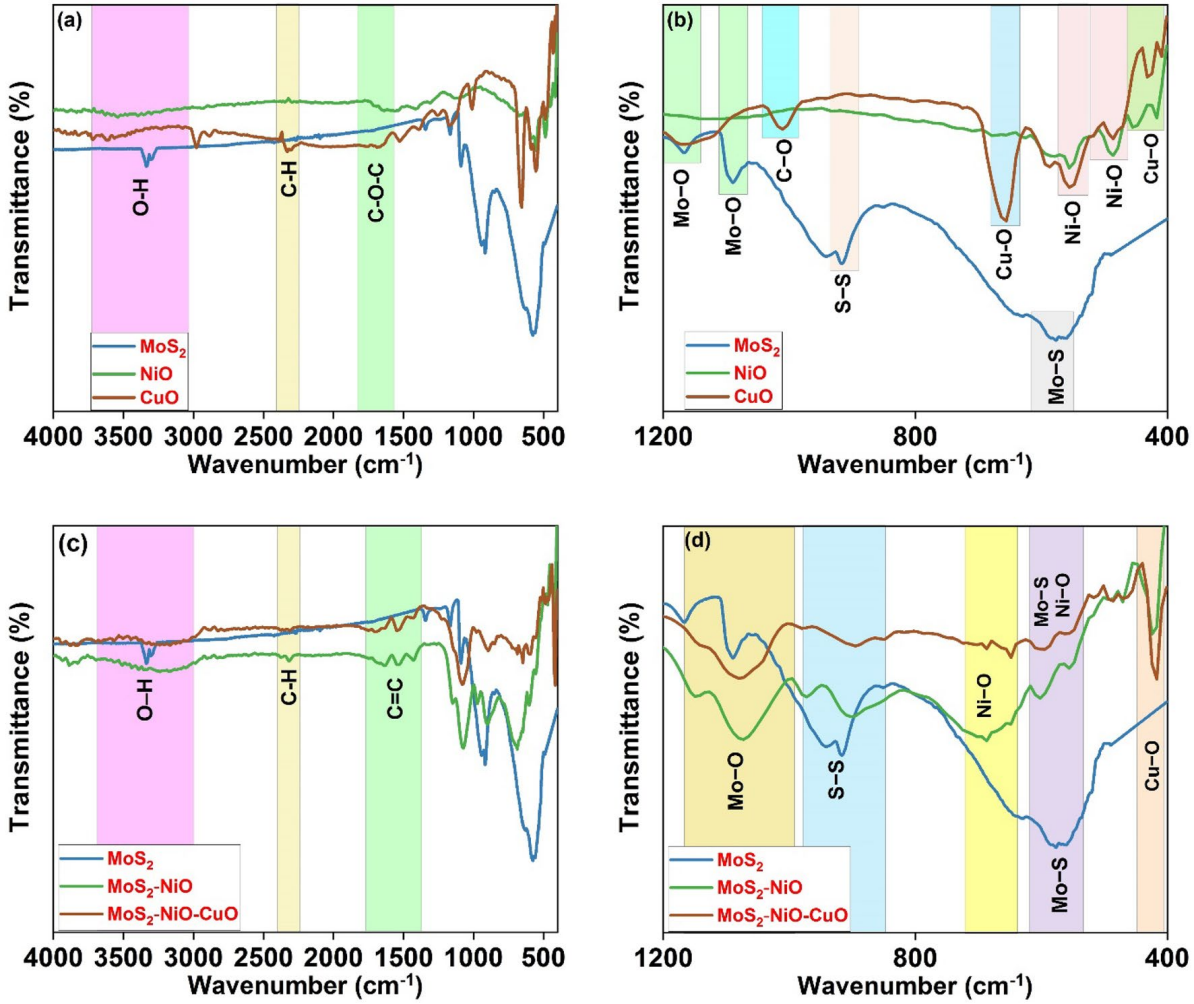
FTIR spectra (**a**) wave number between 4000 to 400 cm^−1^, (**b**) enlarged wave number between 1200 to 400 cm^−1^ for pure MoS_2_, NiO and CuO; (**c**) wave number between 4000 to 400 cm^−1^, (**d**) enlarged wave number between 1200 to 400 cm^−1^ for pure MoS_2_, MoS_2_–NiO and MoS_2_–NiO–CuO nanohybrid.

The FTIR spectra were recorded to confirm the presence of components inside the binary and ternary composites, and the findings are shown in Fig. 2a,c at 4000 to 400 cm^−1^ and the particular wave number between 1200 to 400 cm^−1^ are shown in Fig. 2b,d. The FTIR spectra reveal the unique bands produced by the elemental bonds along with the stretching and forming vibrations of Mo–S, Ni–O, and Cu–O in the samples. From the FTIR spectra of MoS_2,_ the band at 565 cm^−1^ produced from the data indicates the vibration of the Mo–S bond^29^. In contrast, the S–S bond produces a band at 916 cm^−1^, while the Mo–O stretching vibrations produce bands at 1088 and 1167 cm^−1^, respectively. The stretching modes of the Ni–O and Cu–O metal–oxygen bonds are associated with the peaks at 485 and 556 cm^−1^, respectively^30^. Bands characteristic of MoS_2_, NiO, and CuO may be seen in the binary composites of MoS_2_–NiO, as well as in the MoS_2_–NiO–CuO nanohybrid. However, the MoS_2_ peaks are more prominent than the NiO and CuO addition ratios. Assigned to the O–H group, C–H, C=C, and C–O–C stretching vibrations, have peaks that occur^31,32^ at 3000–3750, 2333–1647, 1531–1306, and 3000–3750 cm^−1^, respectively. According to the FTIR analysis, the ternary MoS_2_–NiO–CuO nanohybrid contains all of the peaks, indicating that the heterojunction nanohybrid was successfully constructed.

### XPS analysis

**Figure 3:**
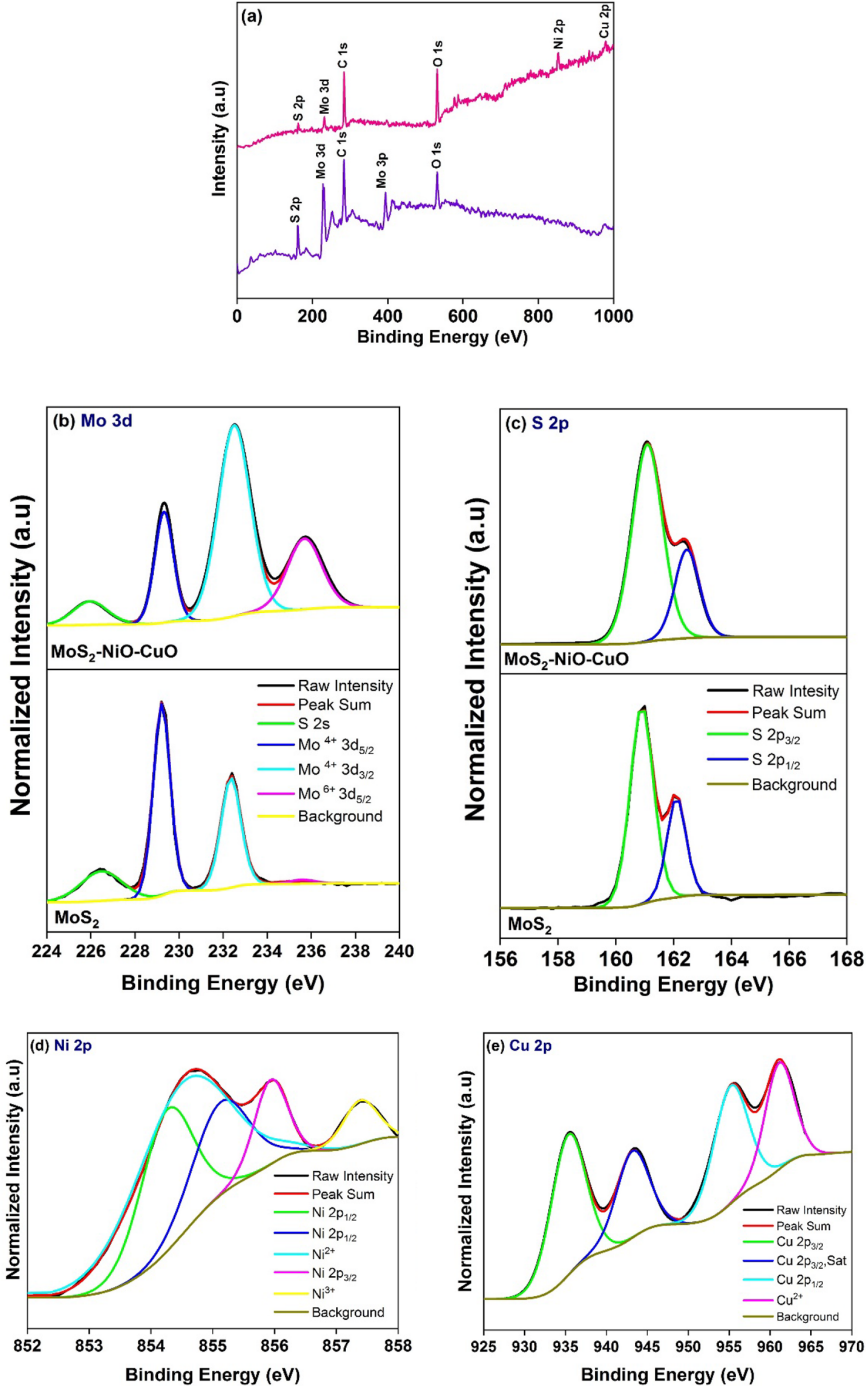
XPS spectra MoS_2_ and MoS_2_–NiO–CuO nanohybrid (**a**) survey spectrum; (**b**) Mo 3d; (**c**) S 2p; (**d**) Ni 2p; (**e**) Cu 2p.

The surface composition and oxidation states of the synthesized samples were further investigated by XPS investigation. The high-resolution XPS survey spectra of the MoS_2_ and MoS_2_–NiO–CuO nanohybrid are shown in Fig. 3a as are presented elements of Mo 3d, S 2p, Cu 2p, Ni 2p, O 1s, and C 1s. Figure 3b shows the Mo 3d spectra of MoS_2_ and MoS_2_–NiO–CuO nanohybrid. The Mo 3d spectra of pure MoS_2_ exhibited the four typical peak at 226.43, 239.23, 232.34, and 235.59 eV attributed to sulfur 2s state (S 2s), Mo in +4 oxidation state (Mo^4+^ 3d_5/2_), Mo in +4 oxidation state (Mo^4+^ 3d_3/2_) and Mo in +6 oxidation state (Mo^6+^ 3d_5/2_) and have been in agreement with the XPS data in the earlier report^33^. In the ternary hybrid, the Mo 3d spectra exhibited the characteristic peak at 226.96 eV attributed to the sulfur 2s state (S 2s), and the three characteristic peaks 239.33, 232.49, and 235.65 eV were assigned binding energies of quad-valent molybdenum (Mo^4+^ 3d_5/2_/Mo^4+^ 3d_3/2_) and hexavalent molybdenum (Mo^6+^ 3d_5/2_). Comparing the binding energies of Mo 3d in the Mo^4+^ 3d_3/2_ state of pure MoS_2_ and MoS_2_–NiO–CuO nanohybrid show that the as-prepared MoS_2_–NiO–CuO nanohybrid has higher binding energies by 0.1 eV. The S 2p spectrum of MoS_2_ and MoS_2_–NiO–CuO nanohybrid is shown in Fig. 3c. In the pure MoS_2_, two peaks of binding energies were observed at 162.5 and 162.85 eV, which were assigned to S 2p_3/2_ and S 2p_1/2_. In the ternary hybrid, S 2p spectrum, the peaks at 161.1 and 162.45 eV correspond to S 2p_3/2_ and S 2p_1/2_, respectively^34^. In the Ni 2p spectrum (Fig. 3d) of the MoS_2_–NiO–CuO nanohybrid, the deconvoluted five peaks at binding energies of 854.30, 854.72, 855.2, 855.97, and 857.44 eV belong to the high binding energy sides of Ni 2P_1/2,_ bivalent nickel (Ni^2+^), Ni 2P_3/2_ and trivalent nickel (Ni^3+^) in NiO, respectively^35^. Figure 3e shown in the Cu 2p spectra of the MoS_2_–NiO–CuO nanohybrid, shows that the binding energies at 935.64, 943.66 and 955.67 eV belong to Cu 2p_3/2,_ Cu 2p_3/2_ (sat) and Cu2 p_1/2_, respectively. The satellite characteristic peak at 961.24 eV assigned to Cu^2+^, confirms that the doped CuO in MoS_2_–NiO is in the Cu^+^ state^36^. In the O 1s spectra of MoS_2_ and MoS_2_–NiO–CuO nanohybrid shown in Fig. S.1 (a). In the O 1s spectra of MoS_2_, three peaks for the O 1s are observed at 530.57, 531.54, and 532.65 eV, whose characteristic peaks correspond to binding energies in MoO, O 1s, and MoO_3_. The O 1s spectra of the MoS_2_–NiO–CuO nanohybrid are shown in Fig S.1. (a), four peaks for the O 1s are observed at 530.98, 531.81, 532.9, and 533.63 eV, which corresponds to the binding energies of MoO, O 1s, NiO, and CuO^37,38^. Finally, Fig. S.1. (b) shows the C 1s XPS spectra of MoS_2_ and MoS_2_–NiO–CuO nanohybrid, the intensive peak at 283.74 and 285.07 eV corresponds to C=C and C–O, respectively^39^. The binding energies of Mo 3d, S 2p, and O 1s were compared with pure MoS_2_ and MoS_2_–NiO–CuO nanohybrid, showing that MoS_2_–NiO–CuO nanohybrid has higher binding energies at 0.1, 0.4 and 0.27 eV. The XPS results strongly confirmed that there were indeed interactions between CuO and MoS_2_–NiO when CuO was loaded onto MoS_2_–NiO to form the MoS_2_–NiO–CuO nanohybrid. The above results suggest that a Z-scheme MoS_2_–NiO–CuO nanohybrid heterojunction has been effectively constructed, and the close interaction between MoS_2_–NiO and CuO is favorable to electron transport, which is helpful for the catalytic process^16,38^.

### Morphology analysis

**Figure 4:**
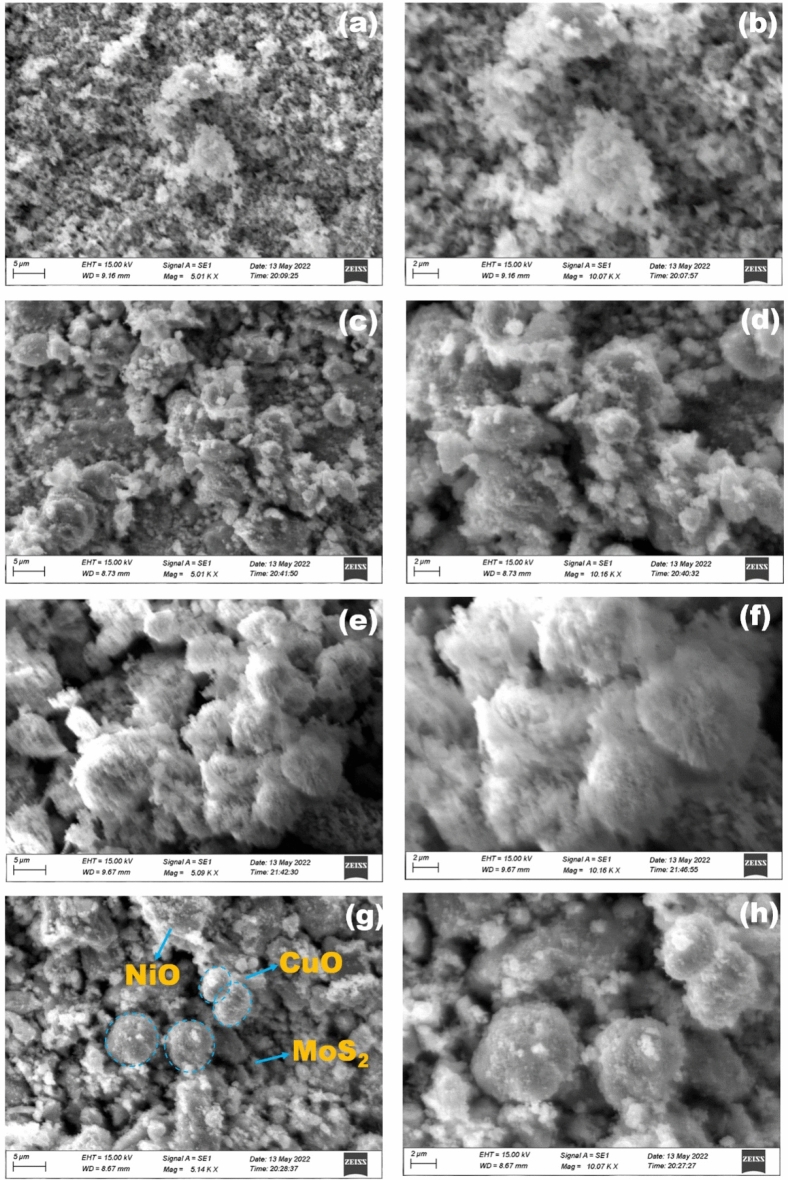
SEM images (**a**,**b**) MoS_2_; (**c**,**d**) NiO; (**e**,**f**) MoS_2_–NiO; (**g**,**h**) MoS_2_–NiO–CuO nanohybrid.

**Figure 5:**
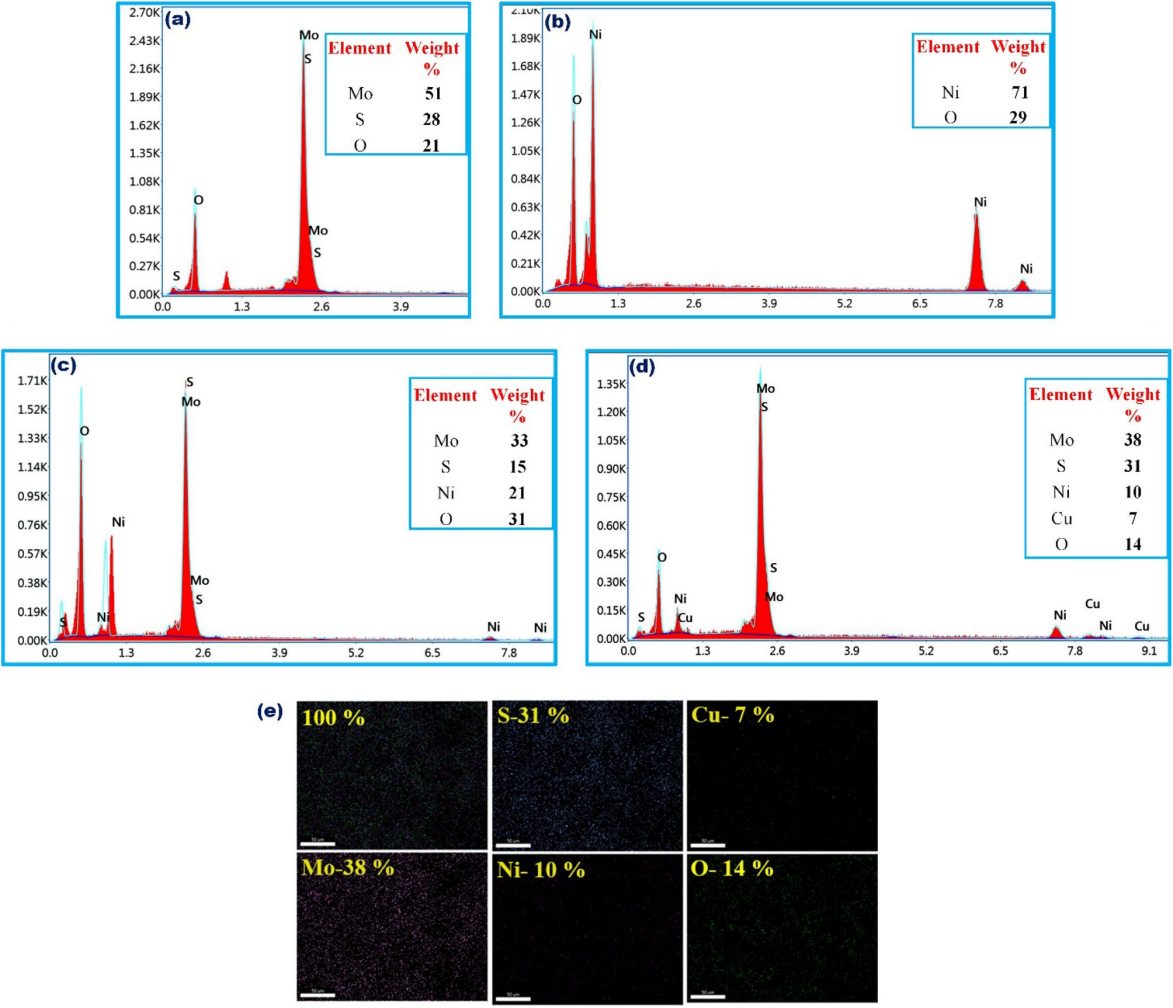
EDAX images (**a**) MoS_2_, (**b**) NiO, (**c**) MoS_2_–NiO, (**d**) MoS_2_–NiO–CuO nanohybrid; (**e**) Elemental mapping and presenting element in % -MoS_2_–NiO–CuO nanohybrid.

The morphology of the synthesized MoS_2_–NiO–CuO nanohybrid was confirmed by scanning electron microscopy (SEM) and transmission electron microscopy (TEM) investigations. The SEM images are shown in Fig. 4, where MoS_2_ is built by a nonuniform nanoparticle (Fig. 4a,b) structure and NiO displays the nanoparticles structure (Fig. 4c,d). The MoS_2_–NiO has a surface morphology that reveals the formation of mixed nanoparticles structure (Fig. 4e,f). The single phase self-growth of CuO nanospheres and MoS_2_ nanoparticles were decorated on the surface of NiO to develop MoS_2_–NiO–CuO nanohybrid heterostructure can be seen in Fig. 4g,h. Following the procedure of adding CuO, the nanosphere arrays have also been successfully retained. Notably, the surface of the ternary MoS_2_–NiO–CuO becomes fairly rough and transparent, in contrast to the smooth surfaces of MoS_2_ and NiO nanoparticles. Figure 5 shows the EDAX spectra of (a) MoS_2_, (b) NiO, (c) MoS_2_–NiO, and (d) MoS_2_–NiO–CuO nanohybrid. The EDAX spectra of pure MoS_2_ the presence of the elements Mo, S, and O which consist of 51, 28, and 21 by weight %. The weight percentages of the Mo, S, Ni, Cu, and O components in the synthesized MoS_2_–NiO–CuO nanohybrid are 38, 31, 10, 7, and 14%, respectively (Fig. 5d). The Fig. 5e displays the element mapping of the as-prepared samples MoS_2_–NiO–CuO nanohybrid. As a result of the discovery of impurities in the synthetic MoS_2_–NiO–CuO nanohybrid, it has been determined that there are no extra elements for the other components, indicating that the synthesized nanohybrid is high purity.

**Figure 6:**
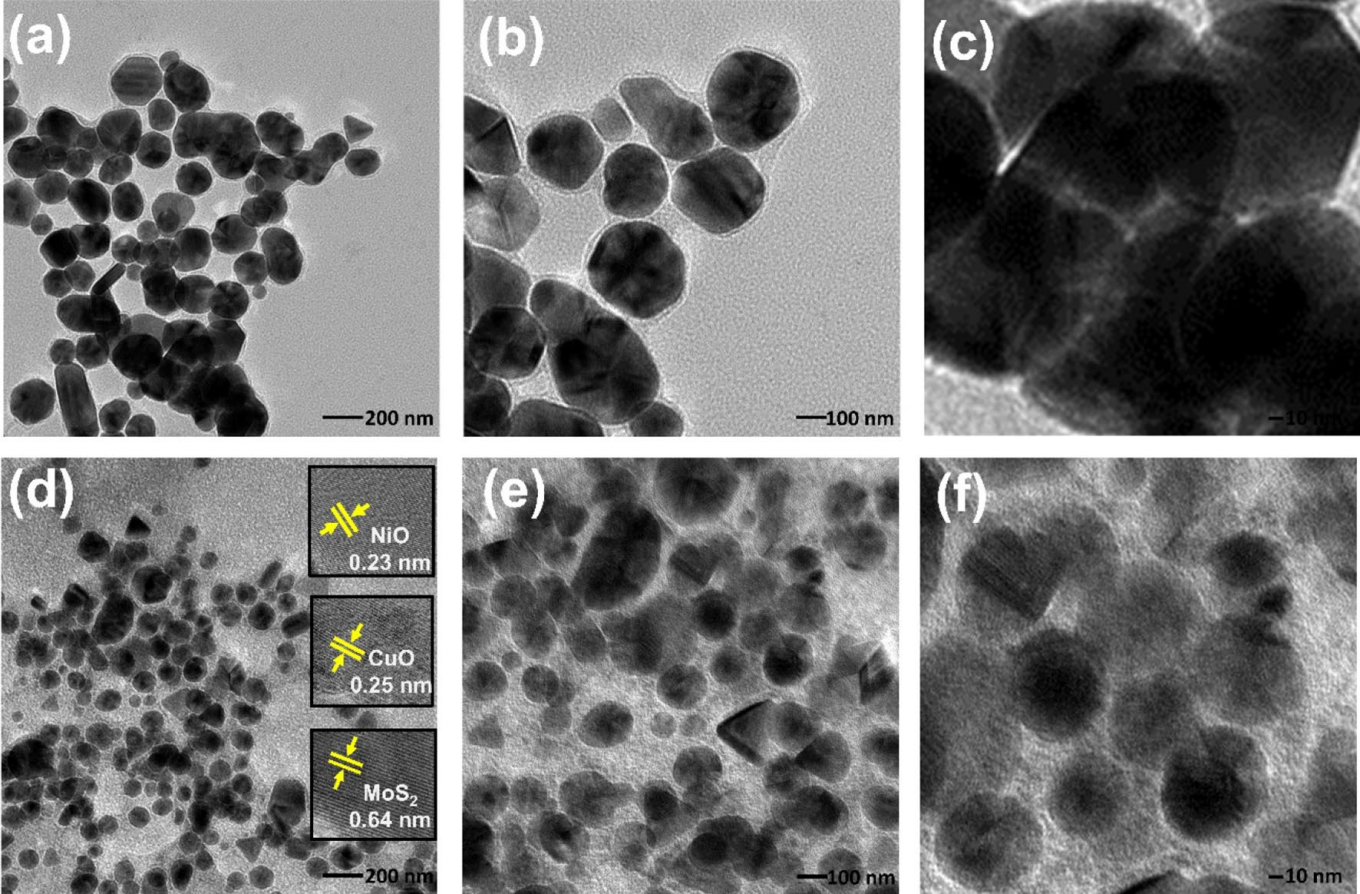
TEM images (**a**–**c**) MoS_2_–NiO; (**d**,**e**) and (**f**) MoS_2_–NiO–CuO nanohybrid, (**d**) insets image lattice fringes.

Figure 6 shows the TEM images of (a–c) MoS_2_–NiO and (d–f) MoS_2_–NiO–CuO hybrid. When the TEM image of MoS_2_–NiO and MoS_2_–NiO–CuO nanohybrid is compared with their SEM images, it is clear that the structure is still intact. The TEM images of MoS_2_–NiO exhibited NiO nanoparticles combined with MoS_2_. In MoS_2_–NiO–CuO nanohybrid, the CuO nanosphere are successfully combined with the MoS_2_ and NiO nanoparticles, then to develop a MoS_2_–NiO–CuO nanohybrid heterostructure as shown in Fig. 6d–f. The observed lattice fringe spacing of 0.64 nm, 0.23 nm and 0.25 nm and these values correspond to the (002), (111) and (111) crystal planes of MoS_2_, NiO and CuO, as shown in Fig. 6d. After the addition of the CuO, it becomes quite clear to observe that the MoS_2_ and the NiO nanoparticles have been completely transferred into the nanosphere formation. The presence of CuO in the interfacial layer that exists in between MoS_2_ and NiO is further evidence that the MoS_2_–NiO–CuO nanosphere structure has been formed. Based on morphological analysis, the MoS_2_–NiO–CuO nanohybrid morphologies provide numerous active site interfaces between MoS_2_–NiO and CuO. So the MoS_2_–NiO–CuO nanohybrid can improve the photovoltaic and photocatalytic capabilities with excellent light response^40^.

### UV and PL analysis

**Figure 7:**
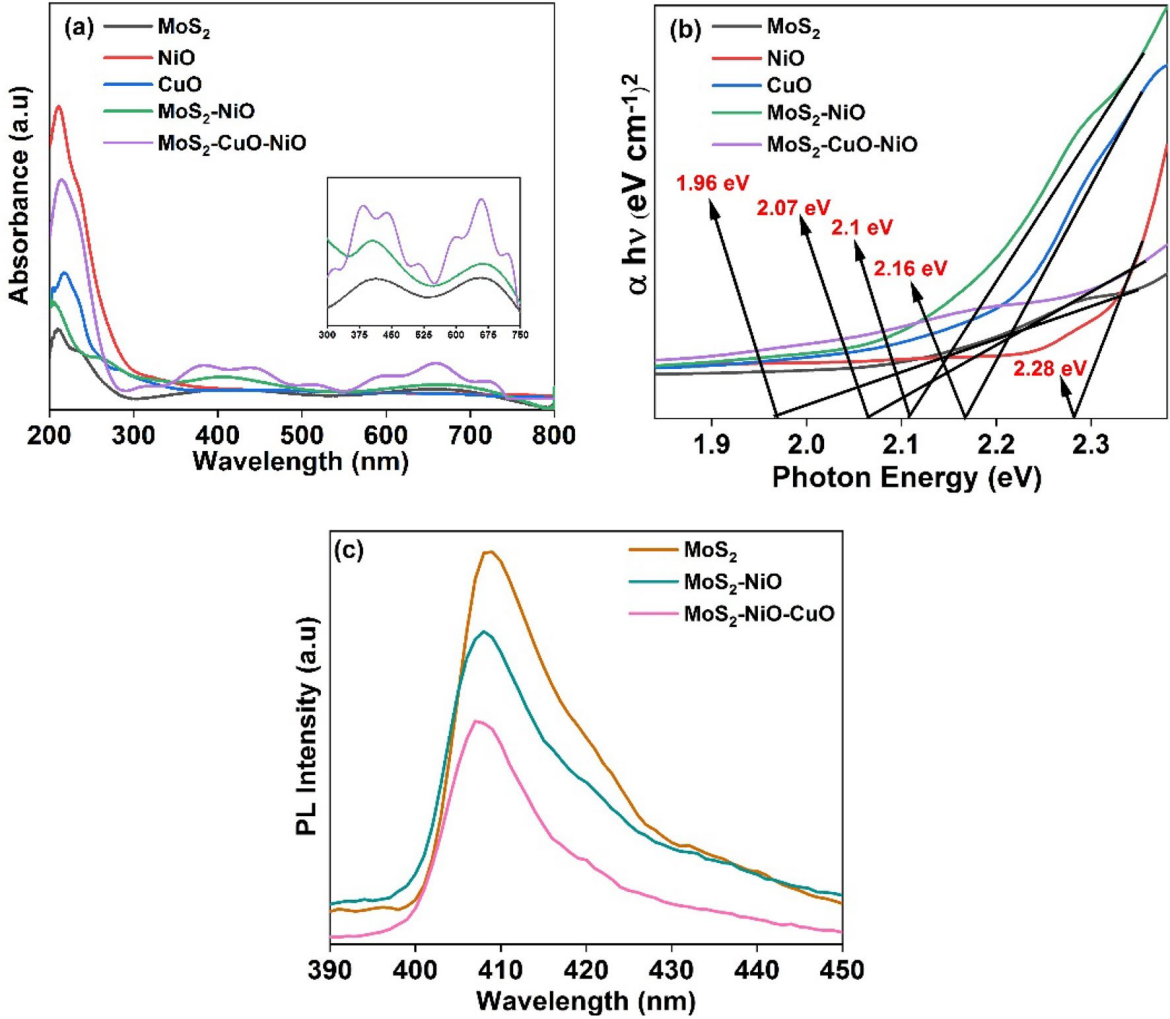
(**a**) UV–Vis spectra; (**b**) Tauc plot; (**c**) PL Spectra of pure MoS_2_, MoS_2_–NiO and MoS_2_–NiO–CuO nanohybrid.

The optical absorption properties of the catalysts are studied by UV–Vis diffuse reflectance spectra. Figure 7a shows that MoS_2_, NiO, and CuO have apparent UV–visible-light absorption peaks at 416 and 662 nm for MoS_2_^41^, 211 and 237 nm for NiO^42^ and 214, 280, and 467 nm for CuO^31^. The enhanced absorbance of the binary composites at 266, 405, and 666 nm for MoS_2_–NiO, indicates that the addition of MoS_2_ improves the efficiency with which NiO uses visible light. Furthermore, the obtained MoS_2_–NiO–CuO nanohybrid exhibited significantly increased visible-light absorption after the addition of CuO at the range between 210 to 720 nm, specifically at 384, 441, and 598, 659, and 719 nm^31^. Absorption of UV–visible light by the ternary material was significantly enhanced in comparison to the binary and ternary composites, with a conspicuous absorption peak observable between 200 and 680 nm in the spectrum. Based on the slope of the tangent line of the plot of (αhν)^2^ vs. photon energy (hν), the band gap energies of MoS_2_, NiO, CuO, MoS_2_–NiO, and MoS_2_–CuO–NiO nanohybrid were determined to be 1.96, 2.28^43^, 2.16, 2.1, and 2.07 eV, respectively as shown in Fig. 7b. UV-DRS results reveal that the adsorption edge is expanded from the UV area to the visible range, leading to greater electron–hole pairs in the ternary composites than in the pure and binary composites. The reduced band gap indicates that the ternary composite increases the conductivity and aids in improved photocatalytic and photovoltaic performance of the MoS_2_–NiO–CuO nanohybrid^44^. Effective charge transfer and separation of the as-prepared heterojunctions may be assessed by measuring the PL spectrum, and the emission produced by the recombination rate of electron–hole pairs. The photocatalytic activity of a given photocatalyst is strongly correlated with the intensity of its photoluminescence. MoS_2_ displays a large and wide emission peak at about 408 nm^45^ with a matching excitation wavelength of 360 nm, corresponding to the rapid recombination rate of photogenerated carriers, as illustrated in Fig. 7c. When MoS_2_–NiO and MoS_2_–NiO–CuO nanohybrid are compared to pure, the PL intensity gradually decreases. Furthermore, the PL intensities for the MoS_2_–NiO–CuO nanohybrid are gradually reduced due to the mixture of CuO, which is indicative that the addition of CuO is aiding in improved separation efficiency and inhibiting recombination of charge carriers at the heterojunctions^45,46^.

### DSSC solar cell performance analysis

**Figure 8:**
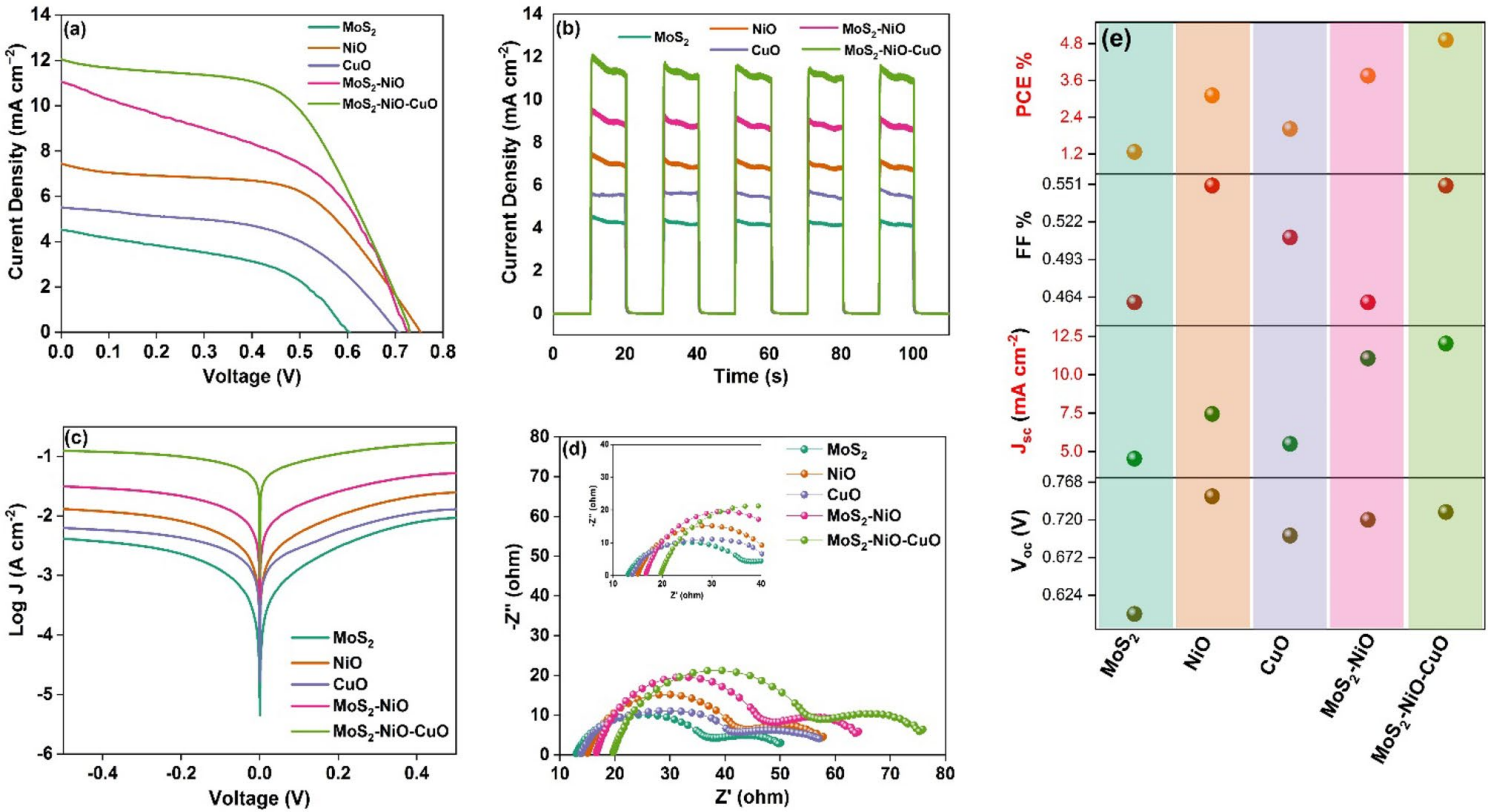
(**a**) Comparative J–V curves; (**b**) Photocurrent response; (**c**) Tafel plot; (**d**) EIS spectrum. (**e**) Comparative DSSC efficiency of MoS_2_, NiO, CuO, MoS_2_–NiO and MoS_2_–NiO–CuO nanohybrid.

Figure 8a represents the photocurrent vs. voltage (J–V) curves of fabricated DSSCs based on various CEs. When N719 dye is used as the sensitizer, the attainable voltage between 0.6 and 0.73 V is shown in the J–V curve. The measured photovoltaic characteristics of the constructed solar cell are presented in Table 1 and these parameters include V_oc_, J_sc_, and fill factor and these values are provided the efficacy of the fabricated DSSC, whose values are contingent upon the concentration of the dye and the proportion of the dye that is absorbed by the photoanode. The calculated efficacy of MoS_2_, NiO, CuO, MoS_2_–NiO, and MoS_2_–NiO–CuO as a result of photovoltaic properties are 1.27, 3.11, 2.01 3.74 and 4.92%, respectively, as shown in Fig. 8e. Interestingly, the MoS_2_–NiO–CuO nanohybrid showed a PCE of 4.92% (V_oc_ = 0.73 mV, Jsc- = 12.04 mA cm^−2^, FF = 0.58%), which is much higher than the efficiency for other samples.

**Table 1:** Photovoltaic parameters of the as fabricated DSSC.

Prepared materials	V_oc_ (V)	J_sc_ (mA cm^−2^)	Fill factor (%)	Efficiency (%)	R_s_ (Ω)	R_ct-1_ Ω	R_ct-2_ Ω
MoS_2_	0.6	4.53	0.46	1.27	19.77	37.68	17.88
NiO	0.75	7.44	0.55	3.11	15.06	27.8	15.7
CuO	0.7	5.5	0.51	2.02	16.58	31.32	16.96
MoS_2_/NiO	0.72	11.06	0.46	3.75	13.92	26.2	16.5
MoS_2_/NiO/CuO	0.73	12.04	0.55	4.92	12.85	24.13	12.81

The transient photocurrent response of the constructed DSSC in a standardized investigation for 110s with 10s on and off intervals. For the five transient photocurrent cycles tested to check the stability of the material, the device with MoS_2_–NiO–CuO has the higher current density, as displayed in Fig. 8b. The improved PCE% is due to the improved electrocatalytic action of the MoS_2_–NiO–CuO nanohybrid counter electrode.

The regions with higher potential, zero potential, and lower potential correspond to the diffusion zone, Tafel zone, and polarization zone of the Tafel polarization curve, respectively. The Tafel polarization curve shows the relationship between potential (V) vs logarithmic current density (log J). The diffusion zone estimates the transport of electrode ions and the Tafel zone delivers the catalytic activity of the electrode. The Tafel polarization plot revealed limiting the diffusion current density (J_lim_) and the transfer current density (J_ο_). The Tafel plots of synthesized MoS_2_, NiO, CuO, MoS_2_–NiO, and MoS_2_–NiO–CuO nanohybrid are shown in Fig. 8c.

Electro-impedance spectroscopy (EIS) is a powerful technique employed in the study of DSSCs. This technique allows to investigate the dynamic behavior of the cell’s electrical components by applying a small amplitude sinusoidal perturbation to the system and analyzing the resulting current response. The electrochemical behavior and charge transfer processes occurring within the cell. One characteristic feature commonly observed in the EIS spectrum of DSSCs is the presence of two distinctive semicircles, each corresponding to specific electrochemical interfaces within the cell. It was analyzed from 10 mHz to 10 kHz with bias of open circuit voltage, Fig. 8d, the Nyquist plot that was obtained from the EIS spectra is displayed. The high-frequency semicircle typically appears in the EIS spectrum and is associated with the charge transfer resistance (R_ct-1_) at the electrolyte/counter electrode interface. This region represents the impedance encountered during the transfer of electrons between the counter electrode and the electrolyte. A larger semicircle suggests higher charge transfer resistance, potentially indicating limitations in the kinetics of the electrochemical reactions at this interface. The low-frequency semicircle is associated with the interaction between the photoanode and the electrolyte. This region, denoted as R_ct-2_, signifies the charge transfer resistance at the photoanode/electrolyte interface. A detailed analysis of R_ct-2_ aids in understanding the efficiency of charge transfer reactions in this critical region.

The prepared active materials fitted values of R_ct-1_ are MoS_2_ (37.68 Ω), NiO (27.8 Ω), CuO (31.32 Ω), and MoS_2_–NiO (26.2 Ω) for and MoS_2_–NiO–CuO (24.13) nanohybrid, respectively. Hence, the MoS_2_–NiO–CuO nanohybrid displays a lower R_ct-1_ due to the addition of PE and high catalytic activity. The diffused semicircle in the middle region concerns R_ct2_ which describes the charge transfer function of TiO_2_@Dye/electrolyte as MoS_2_ (17.88 Ω), NiO (15.7 Ω), CuO (16.96 Ω), MoS_2_–NiO (16.5 Ω) and MoS_2_–NiO–CuO (12.81 Ω) for nanohybrids. The sheet resistance (R_s_) is dependent on the contact between active material and substrate. In the prepared DSSC devices, it was achieved at 19.77, 15.06, 16.58, 13.92, and 12.85 Ω for MoS_2_, NiO, CuO, MoS_2_–NiO, and MoS_2_–NiO–CuO nanohybrid respectively. Table 2 displays the results of comparing the efficacy of DSSC of the current work to that of earlier works^47–58^. Overall, the findings of comparing the efficacy of CE of DSSC indicate that the as-synthesized MoS_2_–NiO–CuO ternary hybrid has a higher efficacy.

**Table 2:** Comparative behavior of DSSCs with different hybrid counter electrodes.

Counter electrodes	V_oc_ (V)	J_sc_ (mA cm^−2^)	FF (%)	PCE %	References
Co-Mo-S@NG with ETA	0.49	7.22	0.44	1.18	^47^
RGO-Co-MoS	0.39	15.47	0.34	1.55	^48^
NiO@NiS@graphene	0.76	4.86	0.56	2.10	^49^
ZnO/TiO_2_/CdS	0.46	7.8	0.68	2.4	^50^
Cu_2_ZnSnS_4_ (CZTS)	0.46	9.2	0.61	2.65	^51^
ZnS(6)/CIS123–TiO_2_	0.75	8.72	0.53	3.54	^52^
CdS_x_Se_1-x_ Quantum dots-sensitized TiO_2_	0.56	11.85	0.54	3.58	^53^
Polyaniline/single walled carbon nanotube/ZnO	0.71	9.59	0.56	3.81	^54^
CdSe@ CuO/TiO_2_/ZnO	0.73	10.64	0.49	3.81	^55^
Cu_2_ZnSnS_4_/MoS_2_	0.72	8.45	0.66	4.07	^56^
NiCo_2_O_4_/rGO/PANI	0.71	13.9	0.48	4.67	^57^
Ni_65_–PANI_30_–G_5_ (PG3)	0.754	11.36	0.54	4.74	^58^
MoS_2_/NiO/CuO	0.73	12.04	0.55	4.92	Present work

### Photocatalytic activity analysis

A provided UV–visible light source (500-W halogen) is critical in converting degradation products into photosynthetic reactions. There is no change in dye concentration in the absence of a catalyst. However, when a catalyst is used in a photocatalytic reaction, the concentration of dye varies with the capacity of the catalyst produced. The UV–Vis absorption spectra of photodegraded dyes for prepared samples are shown in Fig S.(2) and S.(3), indicating the photocatalytic performance of the dyes produced by the photocatalyst by (1) CV and (2) MO dye degradation at 80 and 100 min, respectively. The UV–Vis absorption spectra presented in Fig S.(2) and S.(3) show maximum peak positions at 586 nm and 465 nm corresponding to the CV and MO dye, respectively. The absorbance intensity of CV and MO dye with photocatalyst decreases with increasing time of UV–Vis light irradiation.

**Figure 9:**
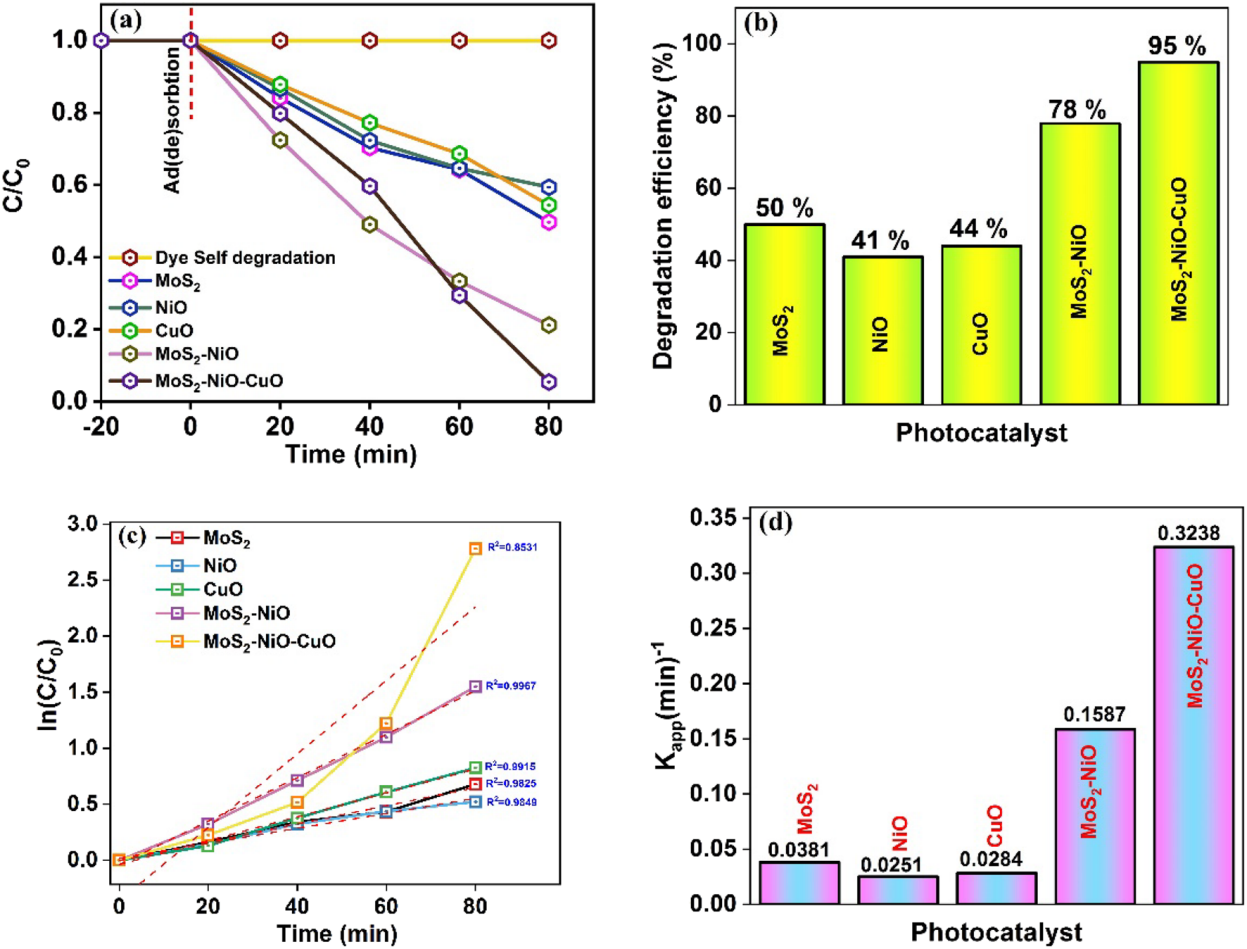
(**a**) (C/C_0_) vs irradiation time plot, (**b**) photocatalytic degradation efficiency for CV dye. (**c**) plots of ln (C/C_0_) vs irradiation time (Pseudo first order kinetics equation), (**d**) highest Rate constant (k) for CV Dye.

The self-degradation process of CV dye is studied without using any catalysts, and it is found that there is no significant change in CV dye concentrations. Figure 9a shows the dye concentration (C/C_0_) versus irradiation time, which identified the photocatalytic efficiency of the different prepared photocatalysts, viz., MoS_2_, NiO, CuO, MoS_2_–NiO and MoS_2_–NiO–CuO nanohybrid. The produced MoS_2_, NiO, CuO, MoS_2_–NiO and MoS_2_–NiO–CuO nanohybrid efficiencies for CV dye were 50, 41, 44, 78, and 95% at 80 min, respectively, as shown in Fig. 9b. The linear relationship between the ln (C/C_0_) vs irradiation time (t) for CV dye degradation is depicted in Fig. 9c. The pseudo-first order kinetics of MoS_2_, NiO, MoS_2_–NiO, and MoS_2_–NiO–CuO nanohybrid of CV dye were studied, and the calculated rate constant (k_1_) values are 0.0381, 0.0251, 0.0284, 0.1587 and 0.3238 min^−1^, respectively. The rate constant of the MoS_2_–NiO–CuO nanohybrid was 8.5 times higher than that of MoS_2_ as shown in Fig. 9d.

**Figure 10:**
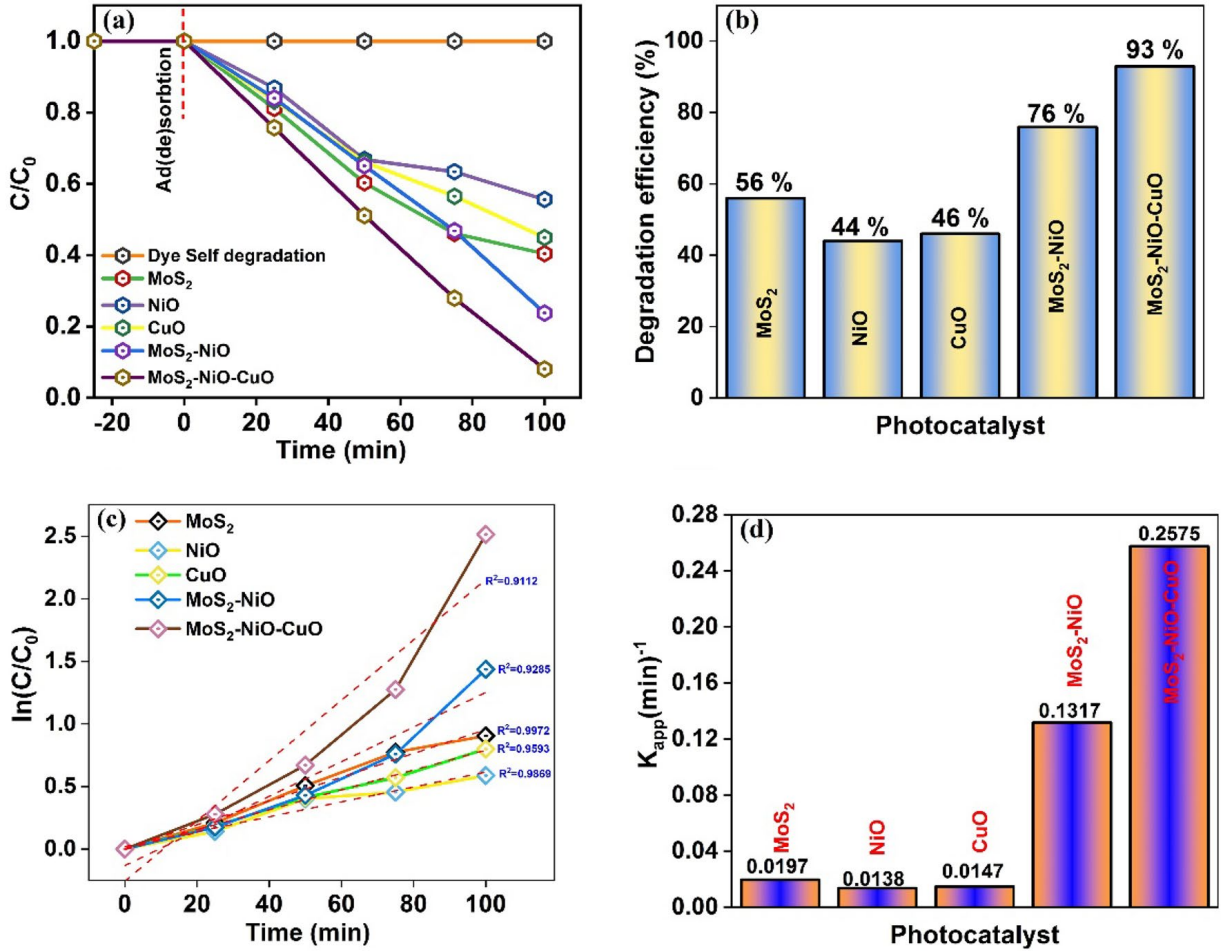
(**a**) (C/C_0_) vs irradiation time plot, (**b**) photocatalytic degradation efficiency for MO dye, (**c**) plots of ln (C/C_0_) vs irradiation time (Pseudo first order kinetics equation), (**d**) highest Rate constant (k) for MO Dye.

A similar self-degradation of the MO dye is detected that there is no remarkable change in concentration of the MO dye and the relative dye concentration (C/C_0_) vs time curve is shown in Fig. 10a. The partially MO dye decolorized after 100 min of irradiation and the highest degradation efficiency of MoS_2_–NiO–CuO was 93% compared to all related samples as shown in Fig. 10b. The linear relationship between the ln (C/C_0_) vs irradiation time (t) for MO dye degradation is shown in Fig. 10c. The pseudo-first order kinetics equations and the kinetics of pure MoS_2_, NiO, CuO, MoS_2_–NiO, and MoS_2_–NiO–CuO nanohybrid of MO dye were studied and the calculated rate constant (k_1_) is 0.0197, 0.0138, 0.0147, 0.1317 and 0.2575 min^−1^, respectively. The rate constant of the MoS_2_–NiO–CuO nanohybrid was 13 times higher than that of pure MoS_2_, which is clear from Fig. 10d. The pseudo-second order kinetics of MoS_2_, NiO, CuO, MoS_2_–NiO, and MoS_2_–NiO–CuO nanohybrid were studied and these results are shown in S.I. (4) (a) for CV and S.I. (4) (b) for MO dyes.

From the overall photocatalysts, the results of both CV and MO dye degradation efficiencies are as follows: NiO < CuO < MoS_2_ < MoS_2_–NiO < MoS_2_–NiO–CuO nanohybrid. Compared to pure and binary photocatalysts, the MoS_2_–NiO–CuO nanohybrid has higher photocatalytic efficiency in the photodegradation of CV and MO dyes. The results show that the charge separation between MoS_2_–NiO and CuO allows for higher photodegradation in the MoS_2_–NiO–CuO nanohybrid, which is formed after adding CuO to MoS_2_–NiO.

**Figure 11:**
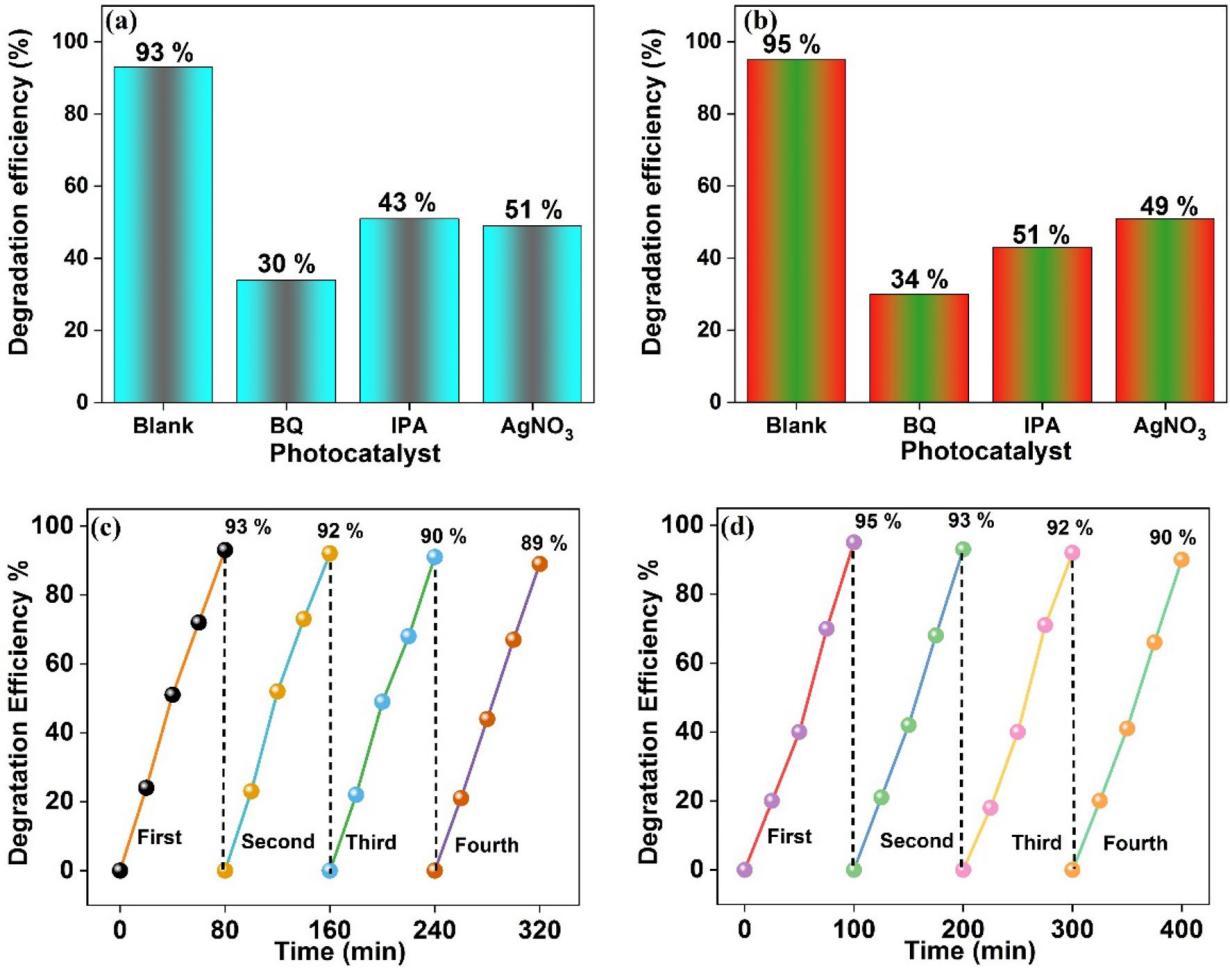
Effects of different scavengers existence of MoS_2_–NiO–CuO nanohybrid (**a**). MO (**b**). CV dye; Recyclability of the MoS_2_–NiO–CuO nanohybrid upto 4th cycles (**c**). MO (**d**). CV dye.

The scavenger test was used to find the most active species involved in the degradation process. The Fig. 11 as illustrates the effects of different scavengers existence of MoS_2_–NiO–CuO nanohybrid by (a) MO and (b) CV dyes. The MO degradation efficiency noticed for various scavengers as follows: BQ = 30%, IPA = 43% and AgNO_3_ = 51%. In case of CV, the degradation efficiency were BQ = 34%, IPA = 51% and AgNO_3_ = 41%. After the addition of BQ to the degradation process, the scavenger experiment shows that both degradation efficiencies are hardly inhibited. The above results suggest that superoxide radicals (^**·**^O_2_^−^) is the most reactive species than ·OH radicals and e^−^ radicals. The ^**·**^O_2_^−^ plays a critical role in the photocatalytic degradation of CV and MO by MoS_2_–NiO–CuO nanohybrid.

**Figure 12:**
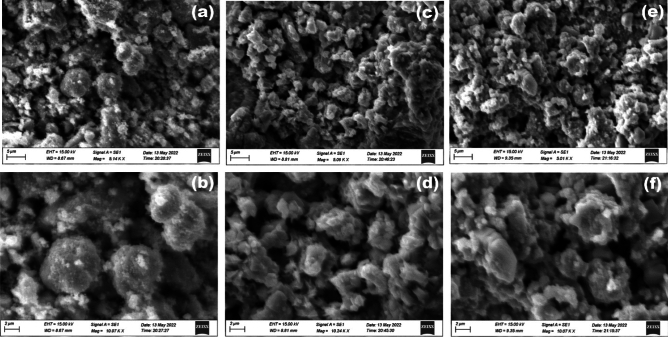
SEM images (**a**,**b**) without dye MoS_2_–NiO–CuO nanohybrid; (**c**,**d**) before and (**e**,**f**) after 4th cycles MoS_2_–NiO–CuO nanohybrid.

**Figure 13:**
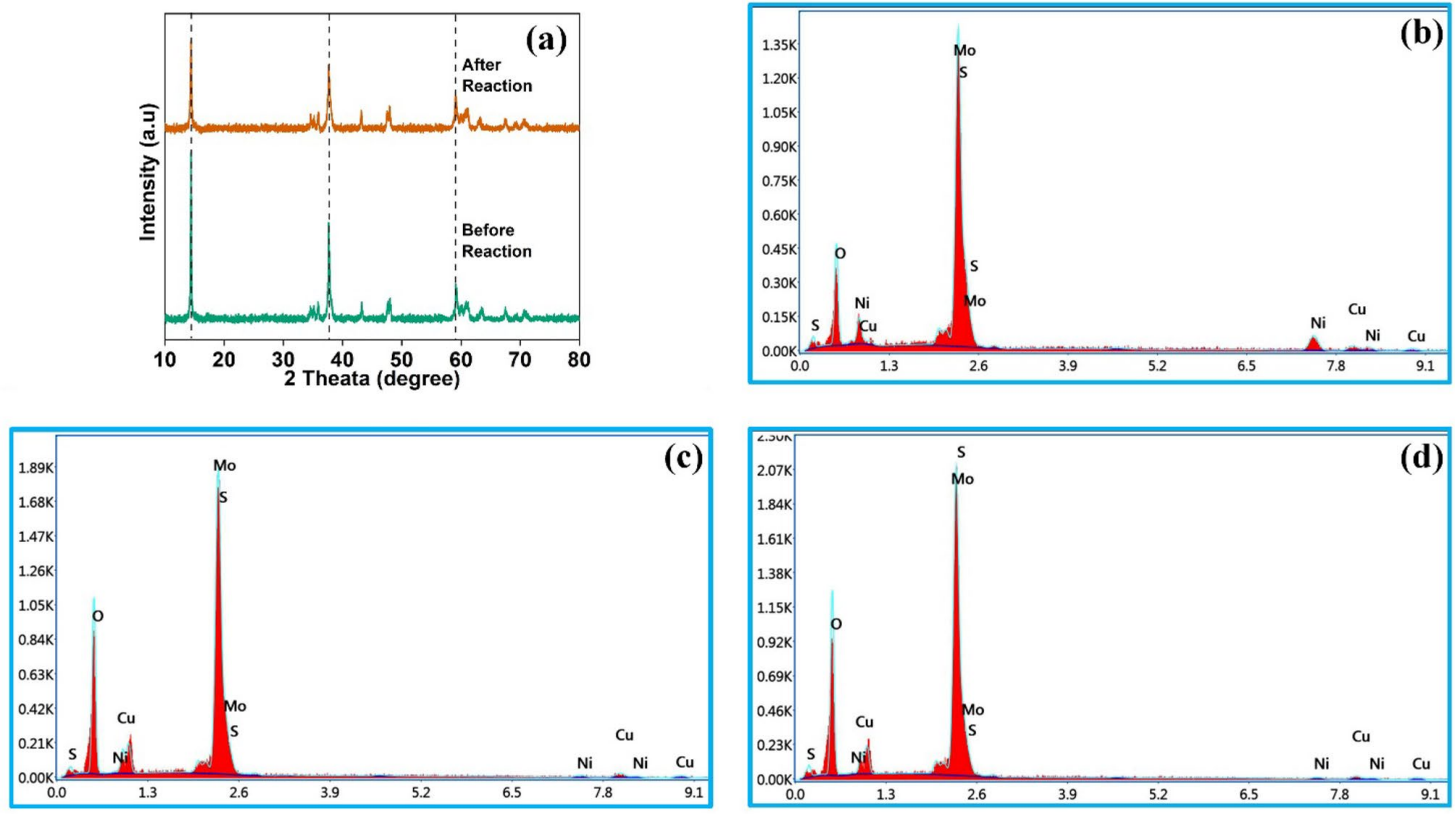
(**a**) XRD Pattern of before and after 4th cycle MoS_2_–NiO–CuO nanohybrid; (**b**) EDAX-pure MoS_2_–NiO–CuO nanohybrid; (**c**) EDAX- before 4th cycle and (**d**) EDAX- after 4th cycle.

The two most crucial elements for the practical use of a catalyst are its reusability and stability. To study the photocatalytic effectiveness of the MoS_2_–NiO–CuO nanohybrid for the degradation of CV and MO, the process was repeated four times. As can be seen in Fig. 11, MoS_2_–NiO–CuO nanohybrid degradation efficiency decreases only slightly, with 90% and 89% of (c) CV and (d) MO degraded, after four cycles of recycling respectively. In addition, after the fourth recycling process, the catalyst was washed, cleaned, and dried in a hot air oven to determine its stability. The stability of the catalyst was analyzed by SEM images as shown in Fig. 12. Before and after the photocatalytic processes, the MoS_2_–NiO–CuO nanohybrid did not undergo any noticeable changes in phase, morphology, and elemental composition as illustrated in Fig. 13 (a) XRD and (b–d) EDAX. From the stability test, no obvious variations in the photocatalytic activity of MoS_2_–NiO–CuO nanohybrid were observed after the decomposition of CV and MO dyes, indicating excellent stability and recyclability of MoS_2_–NiO–CuO nanohybrid. The photocatalytic degradation comparison of crystal violet (CV)^59–65^ and methyl orange (MO) dye^66–72^ by presented work and previous works as shown in Tables 3 and 4. From the table of overall photocatalytic analysis results, the freshly prepared MoS_2_–NiO–CuO ternary hybrid exhibits the most effective degradation performance for CV and MO dyes.

**Table 3:** Comparison photocatalytic degradation of crystal violet (CV) dye by presented work and previous works.

S. no.	Ternary photocatalyst materials	Light source	Used crystal violet (CV) dye (ppm)	Photocatalyst amount (mg)	Irradiation time (min)	Degradation efficiency (%)	References
1	Graphene–Ce-TiO_2_	UV–Visible light	30	6	35	54.2	^59^
2	rGO-ZnS-TiO_2_	UV light irradiation	70	400	30	60.53	^60^
3	rGO-ZnO-TiO_2_	UV light irradiation	50	100	20	87	^61^
4	La_1-x_ Co_x_Cr_1-y_ Fe_y_O_3_/r-GO	Solar irradiation	5	5	90	89	^62^
5	Iron–Bismuth Selenide–Chitosan Microspheres (BISe-CM)	Solar irradiation	50	200	150	94	^63^
6	Fe_3_O_4_/ZnO nanoparticles embedded carboxylate-rich carbon (Fe_3_O_4_/ZnO/CRC)	Solar irradiation	10	75	90	94.5	^64^
7	SnO_2_–Fe_2_O_3_-rGO	UV light irradiation	10	100	120	96	^65^
8	MoS_2_/NiO/CuO	500-W halogen light	20	20	80	95	This work

**Table 4:** Comparison photocatalytic degradation of methyl orange (MO) dye by presented work and previous works.

S. no.	Ternary photocatalyst materials	Light source	Used methyl orange (MO) dye (ppm)	Photocatalyst amount (mg)	Irradiation time (min)	Degradation efficiency (%)	References
1	Au-BiOCl-BiVO_4_	117 W LED lamp	10	250	240	67	^66^
2	Ag/AgCl/g-C_3_N_4_	300 W Xe arc lamp	10	70	180	83.5	^67^
3	Fe_x_O_y_/TiO_2_/Au	90 W LED lamp	10	100	240	90	^68^
4	TiO_2(Anatase)_/WO_3_/TiO_2(Rutile)_	FL15AQ lamps (15 W)	20	500	360	90	^69^
5	Silver tungstate-MoS2-GO	Solar irradiation	10	50	90	93	^70^
6	Ag/C_3_N_4_/RGO	300 W xenon lamp (PLS-SXE300)	10	50	360	93	^71^
7	Ag/AgCl/CaTiO_3_	Xe lamp (300 W, CELHXF300, Beijing)	10	100	50	95	^72^
8	MoS_2_/NiO/CuO	500-W halogen light	20	20	100	93	This work

### Charge transfer mechanism

In contrast, the mechanism of the traditional charge transfer process is different from the Z-scheme heterojunction. A Z-scheme type charge transfer process has been presented in the presence of MoS_2_–NiO–CuO nanohybrid to generate the effective charge separation between the CB of MoS_2_ and the VB of NiO and CuO, which may serve as a feasible approach to increase and extend charge life. Due to the proximity of these two types of semiconductors, P–N junctions appeared on the surface of the p-type semiconductor of MoS_2_ and NiO on the n-type semiconductor^73^. To understand the electron transfer mechanisms of the produced materials, it is necessary to evaluate the reduction and oxidation capacities of electrons and holes within the MoS_2_–NiO–CuO hybrid. The Eqs. (6) and (7) given below can be used to calculate the energy level of the conduction band (E_CB_) and valence band (E_VB_) in the MoS_2_–NiO–CuO hybrid.

6 $$E_{VB} = \chi - E_{e} + 0.5 \;E_{g}$$

7 $$E_{CB} = E_{VB} - E_{g}$$

**Figure 14:**
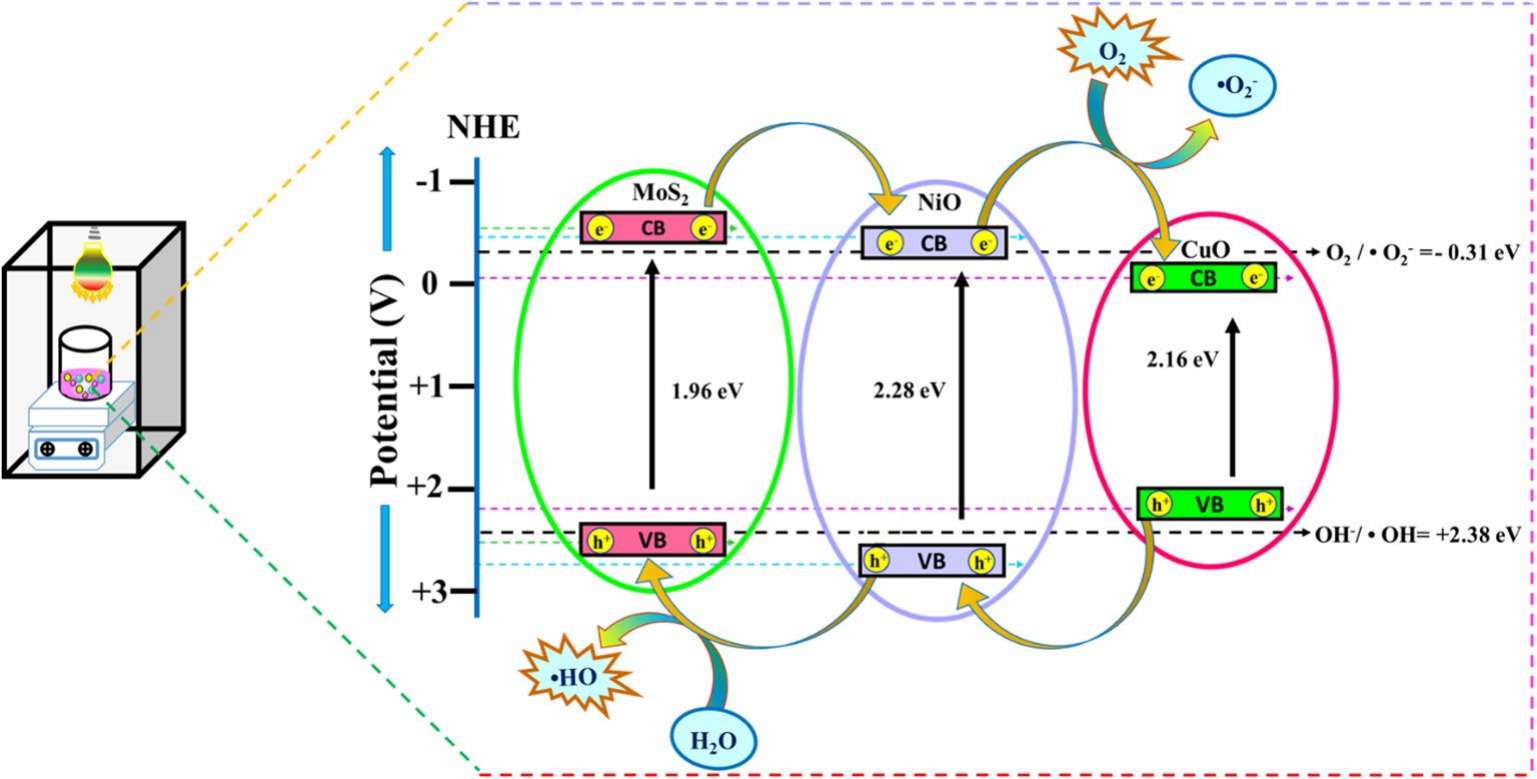
Z-scheme photocatalytic mechanism for organic dye degradation over MoS_2_–NiO–CuO nanohybrid.

Here, the symbols χ, Ee, CB, VB and Eg represent the absolute electronegativity, free electron energy in the hydrogen spectrum (4.5 eV), conduction band, valence band and band gap of the material. The UV-DRS spectra analysis showed that the energy gap values for MoS_2_, NiO and CuO were 1.96, 2.28 and 2.16 eV, respectively. The CB and VB of MoS_2_, NiO and CuO are ~ 2.5 and ~ − 0.54 eV (1.96 eV vs NHE), ~ 2.78 and ~ − 0.5 eV (2.28 eV vs NHE) and ~ 2.29 and ~ − 0.13 eV (2.16 eV vs NHE), respectively. The ensuing impurity energy levels have the potential to narrow the gap concerning the CB of MoS_2_ and the CB of NiO. The integrated electric field may inhibit electrons from the CB of MoS_2_ from immediately migrating to the CB of NiO. In addition, the after introduction of CuO can be oxidized into Cu^2+^ during the photocatalytic dye degradation due to CuO being an excellent co-catalyst between the MoS_2_–NiO^74^. When applying a visible-light-driven field to MoS_2_–NiO–CuO sample, CB of electrons from MoS_2_ would shift the CB of NiO, than electrons from the CB of NiO would transfer into the CB of CuO and onto the Fermi level of Cu. The transfer electrons to Cu^2+^ through the π–π conjugated structure when CuO interacts with water and oxygen to produce Cu^2+^. As a result, Cu^2+^ may be converted to Cu^+^, allowing for the recycling and long-term usage of Cu_2_O^75^. Photo-generated holes through VB of CuO and holes from VB of NiO prefer to transfer to MoS_2_ and after incorporate with photo-generated holes from MoS_2_. The lifetime of the electron in the VB of NiO and the hole in the VB of MoS_2_ is enhanced due to the better conductivity of CuO and its higher positive Fermi energy^76^. Additionally, P–N junctions of MoS_2_–NiO and oxidized CuO contribute to the Z scheme's charge separation efficiency^74^. When dissolved O_2_ is captured by the accumulated photogenerated electrons in the CB of CuO, the resulting ^**·**^O_2_^−^ may oxidize organic dyes to CO_2_ and H_2_O. Meanwhile, the organic dye can be oxidized to H_2_O by the holes contained in the VB of MoS_2_. As a consequence, electrons through the CB of MoS_2_ are more readily absorbed by CuO atoms on MoS_2_, thus improving the separation of photo-generated electrons and holes. As a result, the electron and hole couples caused by light are dispersed throughout the MoS_2_–NiO semiconductors, suggesting that the transfer and efficient separation of charge carriers are greatly facilitated by the presence of CuO^77^. This boosts the Z-scheme-based charge separation efficiency and oxygen evolution activity. The Z-Scheme heterojunction charge transfer mechanism over the MoS_2_–NiO–CuO nanohybrid is shown in Fig. 14. According to the Fig. 14, the photocatalytic mechanism photoinduced electrons and holes displayed greater oxidizing capacity and reducibility, creating more reactive oxidative species (^**·**^O_2_^−^ and ·OH). The main contribution of both ^**·**^O_2_^−^ and ·OH radicals to the degradation process than e^−^ radicals, resulting a removal of CV and MO dye from wastewater and achieving extremely efficient photodegradation. The presences of reactive species caused removal of CV and MO dye as illustrated below Eqs. (8)–(14):

8 $$Catalyst + visible\; light \;(h\nu ) \to Catalyst\; (e_{CB}^{ - } ) + Catalyst \;(h_{VB}^{ + } )$$

9 $$e_{CB}^{ - } + O_{2} \to \cdot O_{2}^{ - }$$

10 $$\cdot O_{2}^{ - } + 2H^{ + } + e_{CB}^{ - } \to H_{2} O_{2}$$

11 $$H_{2} O_{2} + e_{CB}^{ - } \to OH^{ - } + \cdot OH$$

12 $$h_{VB}^{ + } + H_{2} O \to H^{ + } + \cdot OH$$

13 $$h_{VB}^{ + } + H^{ - } \to \cdot OH$$

14 $$\cdot OH + CV\; and \;MO\; dye \;pollutants \to Degratation\; Products$$

The better DSSC and photocatalytic performances of the MoS_2_–NiO–CuO nanohybrid may be attributed to the following influences. (i) Suitable surface morphologies could be modified by the incorporation of metal oxide, and there is no obvious aggregation of the full sphere, which indicates that the substance has a high degree of stability^78^. (ii) The electron transfer rate of the electrode may be increased by the interaction of different components, which in turn encourages the reduction process of I_3_^−^ and the collection of photogenerated electrons in an external circuit. All of these have the potential to prevent photo-generated electrons from recombining, thereby decreasing dark current production and raising J_sc_ in DSSC^79,80^. (iii) Ni and Cu are first-row transition metal ions and they can couple with the sulfur edges of molybdenum to form the strongest chemical bonding, which accelerates efficient proton adsorption and promotes charge transfer between the CuO, NiO, and MOS_2_. In previous reports, the active sulfur atoms on the exposed edges of MoS_2_ increased redox activity and these unsaturated active sulfur atoms could efficiently bind with H^+^ in the solution, which can easily improve the redox activities in the reaction^81–83^. (iv) The significant dual performances might be attributed to the carrier's recombination activities being inhibited. The band gaps of MoS_2_–NiO–CuO nanohybrids are smaller, there are more recombination centers in the Z-scheme heterojunction, and there are more ways for electrons and holes to separate. These factors help separate charges better and absorb more visible light, as well as significantly increasing the photocatalytic and DSSC solar cell performances^84^.

## Conclusion

In summary, we have prepared MoS_2_–NiO–CuO nanohybrid as Z-scheme heterojunction were synthesized by hydrothermal method. The XRD, FTIR, XPS, and FTIR analyses confirmed the construction of the MoS_2_–NiO–CuO nanohybrid. The MoS_2_–NiO–CuO hybrid morphological studies of SEM and TEM images of both showed the same nanospheres shape, and all their elemental components appear in the EDAX spectrum. The MoS_2_–NiO–CuO nanohybrid explored photocatalytic activity are 95 and 93% for CV and MO dye degradation under UV–Vis light and the PCE (%) of as fabricated MoS_2_–NiO–CuO DSSC solar cell is 3.8 times higher than the MoS_2_. The synergistic impact of CuO introducing and NiO nano-grafting to collectively influence MoS_2_ intrinsic intercalation structure was discovered to increase the photocatalytic and photovoltaic activity due to the quick recombination of the photo-induced electron–hole pairs. The ongoing development work of Z-scheme-based MoS_2_–NiO–CuO nanohybrid will improve future applications in the areas of organic pollutant removal and emergent energy storage in a low-cost and eco-friendly manner.

### Supplementary Information

Supplementary Information.

## Data Availability

The datasets used and analyzed during the current study are available from the corresponding author upon reasonable request.
