# Supplementary material for: Direct Z-scheme heterojunction impregnated MoS2–NiO–CuO nanohybrid for efficient photocatalyst and dye-sensitized solar cell
Source: Sci Rep. 2024 Jun 24;14:14518. doi: 10.1038/s41598-024-65163-5 (PMC11196284; doi:10.1038/s41598-024-65163-5)
Supplement: Supplementary file 1 — Supplementary Information. [file 41598_2024_65163_MOESM1_ESM.docx]

**Supplementary Information (SI)**

**Direct Z-scheme heterojunction impregnated MoS_2_-NiO-CuO nanohybrid for efficient photocatalyst and dye-sensitized solar cell**

Karthigaimuthu Dharmalingam^1^, Arjun Kumar Bojarajan^2,3^, Ramalingam Gopal ^4^, Elangovan Thangavel^1*^, Salah Addin Burhan Al Omari^2^, Sambasivam Sangaraju^3*^

*^1^Smart Energy Materials Research Laboratory, Department of Energy Science and Technology Periyar University Salem, India.*

*^2^Department of Mechanical and Aerospace Engineering, United Arab Emirates University, Al Ain-15551, UAE.*

*^3^National Water and Energy Center, United Arab Emirates University, Al Ain - 15551, UAE.*

*^4^Quantum Materials Research Lab (QMRL), Department of Nanoscience and Technology. Alagappa University, India.*

Corresponding authors: elangoes@periyaruniversity.ac.in; s_sambasivam@uaeu.ac.ae

**Synthesis of NiO**

In a typical synthesis of NiO, 5.2 g of Ni(NO_3_)_2_.6H_2_O were dissolved in 40 mL of DI, and it was allowed to stir for 1 h. The pH of the solution was then adjusted from 6 to 11 with ammonia solution. The dark blue mixture was then transferred to a 100 mL autoclave, which was sealed and heated to 180˚C for 6 h. After the autoclave was cooled, the resulting blue precipitate was separated by centrifugation, washed twice times with DI and ethanol. Then dried in a hot air oven at 80º C for 12 h. The dried product was then calcinated at 400˚C for 4 h, the dark blue color finally changed into black powder NiO.

**Synthesis of CuO**

1.5 g of cupric nitrate and 1.6 g NaOH were mixed with 40 mL of DI and the measured pH value is 14. After that, 1.5 mL of PE extracta (with and without) was added to the above solution, followed by stirring for 1 hour, and the mixture was hydrothermally treated at 150 ºC for 2 hours. The suspension was, centrifuged and dried at 80 ºC for half a day, and the dried product was calcined at 400 ºC for 4 hours. Finally, dark brown CuO and CuO-PE were collected.

**Figures:**


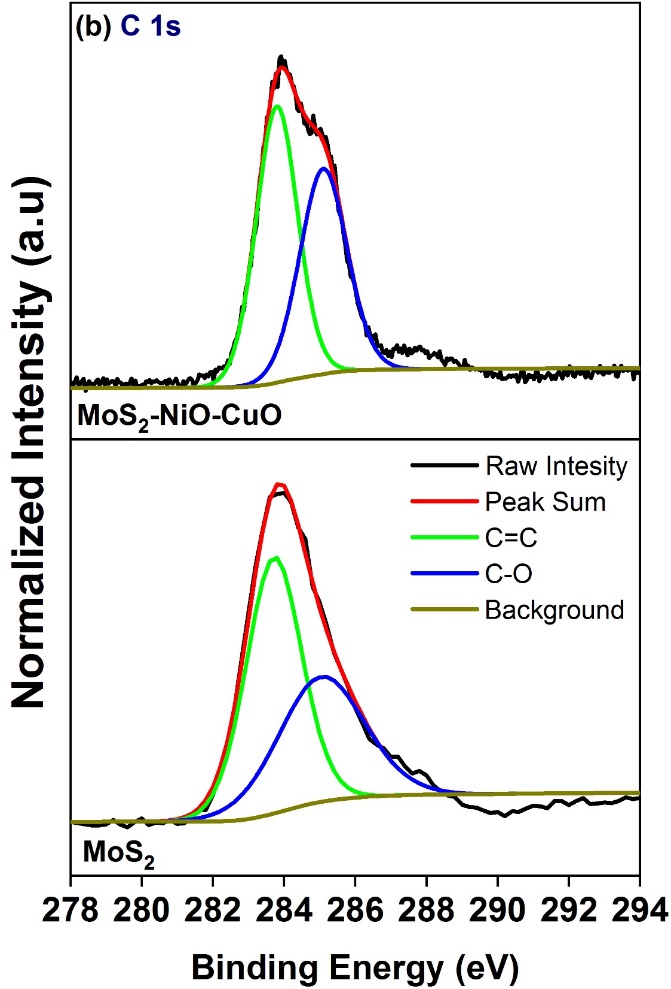

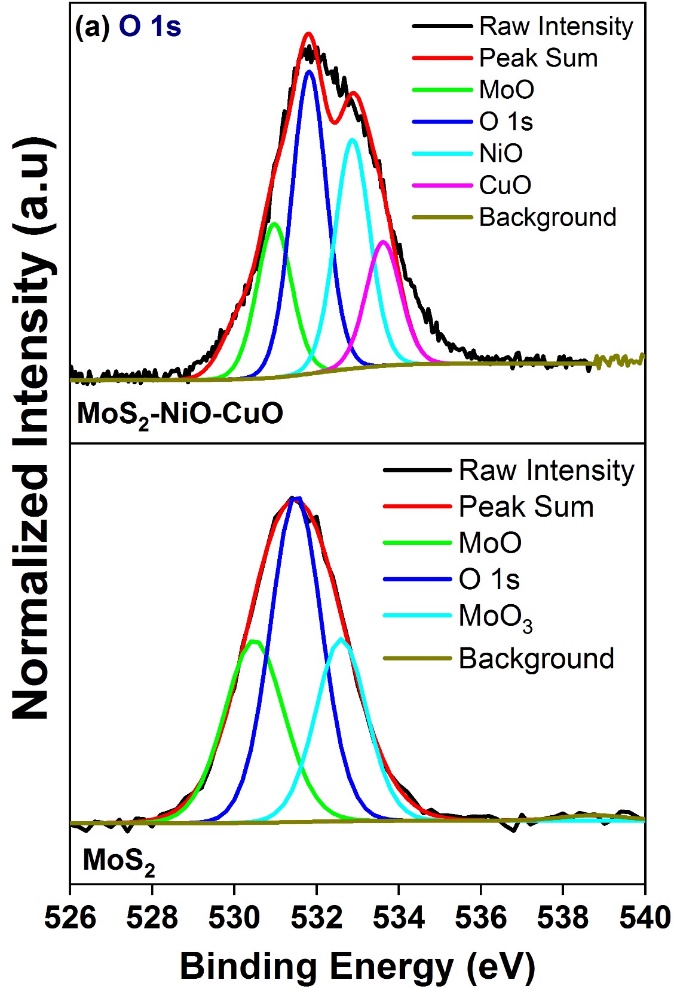


**Fig. S.I.1.** **(a)** O 1s and **(b)** C 1s spectra of MoS_2_ and MoS_2_-NiO-CuO nanohybrid


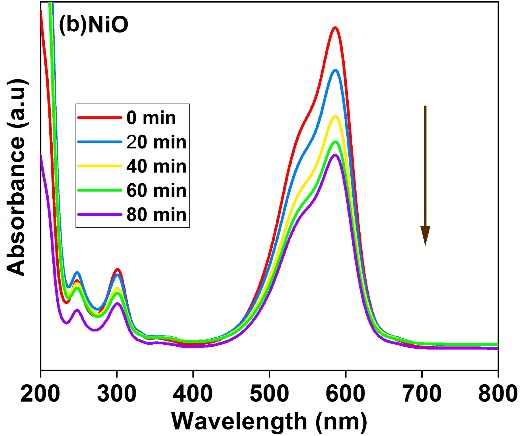

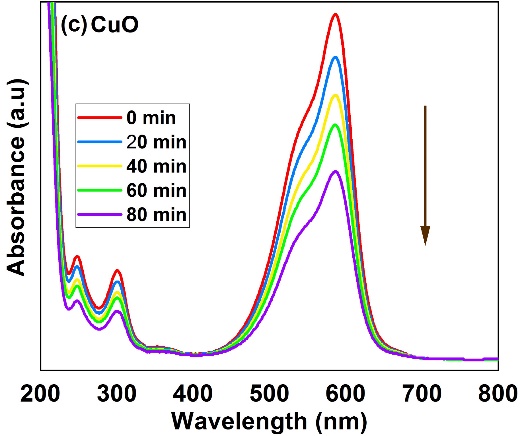

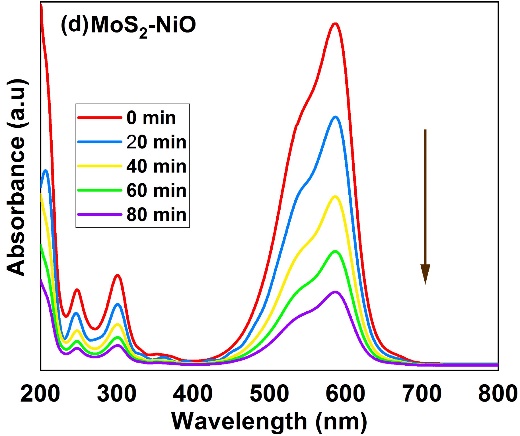

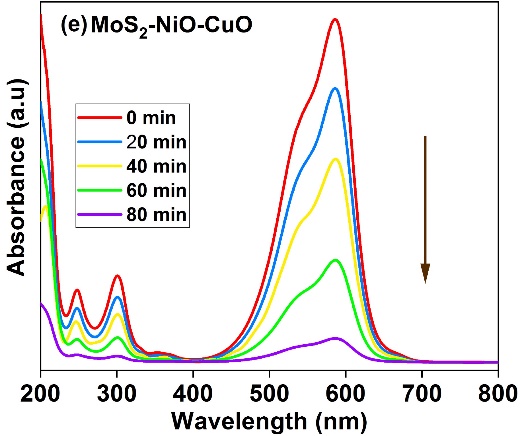

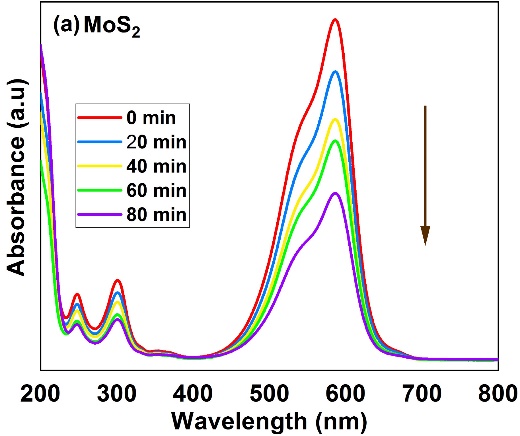


**Fig. S.I.(2).** Photocatalytic degradation UV spectra for CV (Crystal Violet) dye under UV-Vis irradiation


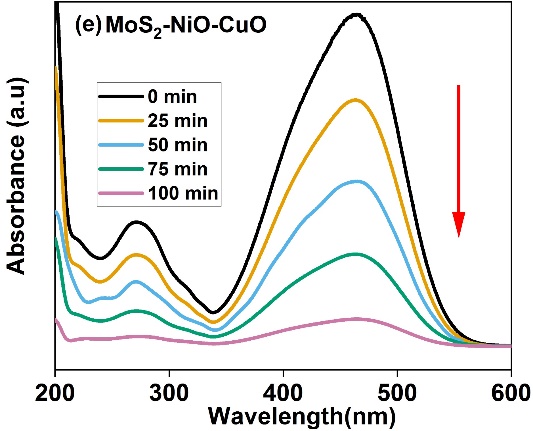

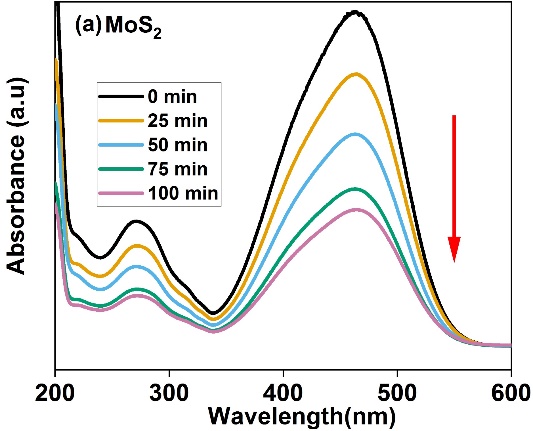

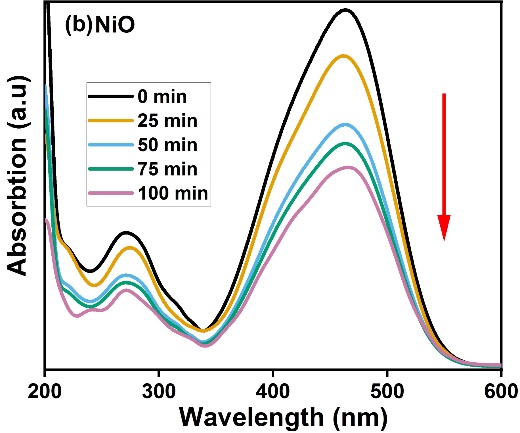

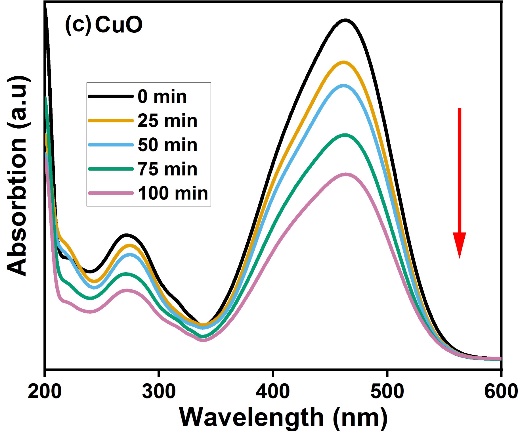

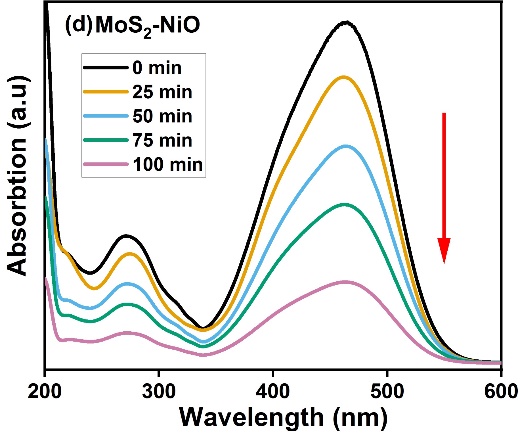


**Fig. S.I. (3).** Photocatalytic degradation UV spectra for MO (Methylene Orange) dye under UV-Vis irradiation


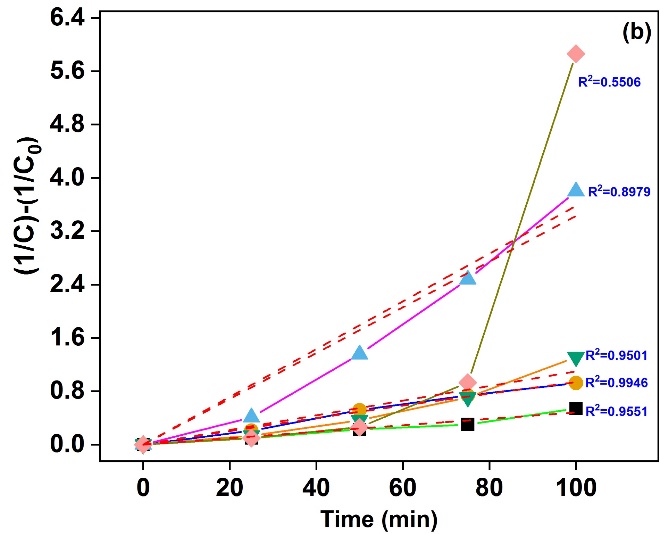

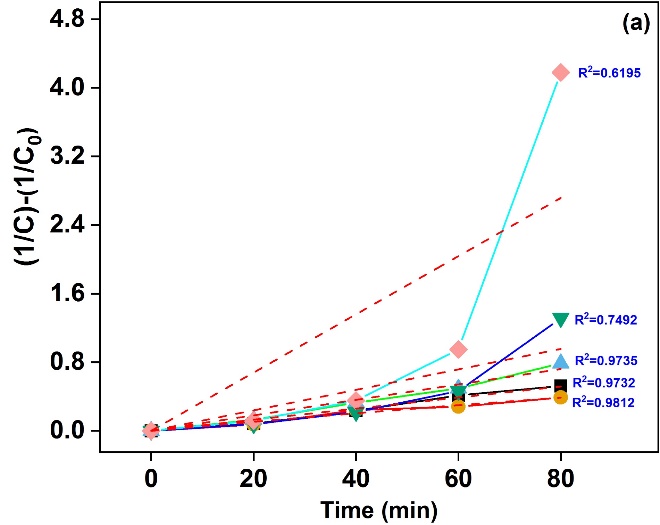


**Fig. S.I. (4).** Plots of 1/C-1/C_0_ vs irradiation time (Pseudo second order kinetics equation) **(a)** for CV Dye and **(b)** for MO Dye
